# Heavy Atom Effect in Halogenated mCP and Its Influence on the Efficiency of the Thermally Activated Delayed Fluorescence of Dopant Molecules

**DOI:** 10.1021/acs.jpcc.3c05567

**Published:** 2024-01-17

**Authors:** Alexandre Malinge, Shiv Kumar, Dongyang Chen, Eli Zysman-Colman, Stéphane Kéna-Cohen

**Affiliations:** † Department of Engineering Physics, École Polytechnique de Montréal, PO Box 6079, succ. Centre-Ville, Montreal QC H3C 3A7, Canada; ‡ Organic Semiconductor Centre, EaStCHEM, School of Chemistry, University of St Andrews, St Andrews, Fife KY16 9ST, United Kingdom

## Abstract

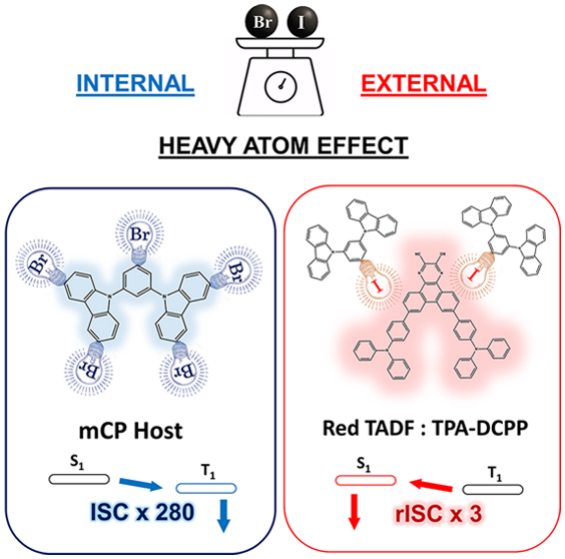

In this study, we explore the impact of halogen functionalization on the photophysical properties of the commonly used organic light-emitting diode (OLED) host material, 1,3-bis(*N*-carbazolyl)benzene (mCP). Derivatives with different numbers and types of halogen substituents on mCP were synthesized. By measuring steady-state and transient photoluminescence at 6 K, we study the impact of the type, number, and position of the halogens on the intersystem crossing and phosphorescence rates of the compounds. In particular, the functionalization of mCP with 5 bromine atoms results in a significant increase of the intersystem crossing rate by a factor of 300 to a value of (1.5 ± 0.1) × 10^10^ s^–1^, and the phosphorescence rate increases by 2 orders of magnitude. We find that the singlet radiative decay rate is not significantly modified in any of the studied compounds. In the second part of the paper, we describe the influence of these compounds on the reverse intersystem crossing of the 7,10-bis(4-(diphenylamino)phenyl)-2,3-dicyanopyrazino-phenanthrene (TPA-DCPP), a TADF guest, via the external heavy atom effect. Their use results in an increase of the reverse intersystem crossing (RISC) rate from (8.1 ± 0.8) × 10^3^ s^–1^ for mCP to (2.7 ± 0.1) × 10^4^ s^–1^ for mCP with 5 bromine atoms. The effect is even more pronounced for the mCP analogue containing a single iodine atom, which gives a RISC rate of (3.3 ± 0.1) × 10^4^ s^–1^. Time-dependent DFT calculations reveal the importance of the use of long-range corrected functionals to predict the effect of halogenation on the optical properties of the mCP, and the relativistic approximation (ZORA) is used to provide insight into the strength of the spin–orbit coupling matrix element between the lowest-lying excited singlet and triplet states in the different mCP compounds.

## Introduction

The ability to harness triplet excited states, for which radiative decay is typically spin-forbidden, is essential to realizing efficient organic luminescent devices such as in phosphorescent or thermally activated delayed fluorescent (TADF) organic light-emitting diodes (OLEDs).^1,2^ These OLEDs can achieve internal quantum efficiencies (IQEs) of up to 100%, as compared to a maximum IQE of only 25% for conventional fluorescent OLEDs, which are limited by the 1:3 singlet:triplet spin-state formation. In both cases, spin–orbit coupling (SOC) is the mechanism driving formally spin-forbidden transitions between singlet and triplet excited states—forward and reverse intersystem crossing (ISC and RISC)^3^—and efficient phosphorescence due to the mixing of states of different spin multiplicity.

Spin–orbit coupling is a relativistic effect resulting from the interaction between the magnetic moment *s⃗* of an electron and its orbital angular momentum *l⃗*. The spin–orbit interaction Hamiltonian for a system with *n* electrons and *N* nuclei is given by^4,5^
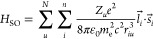
where *Z*_*u*_ is the charge of the nucleus *u*; *m*_e_ is the mass of the electron; *c* is the speed of light in vacuum; *e* is the electron charge; ε_0_ is the vacuum permittivity; and *r*_*iu*_ is the distance between the electron and the nucleus.

For a hydrogenic atom with quantum numbers *n* and *l*, the spin–orbit coupling energy can be estimated using hydrogenic wave function as
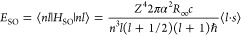
where α is the fine structure constant; *R*_∞_ is the Rydberg constant; and ℏ is the reduced Planck constant.

Given that spin–orbit interaction energy is proportional to the fourth power of the nuclear charge, the vast majority of phosphorescent materials used in luminescent devices have been complexes based on transition metals such as platinum, iridium, or europium.^6−10^ The use of relatively heavy halogen atoms can be a promising alternative to transition metals to increase spin–orbit coupling. As further motivation, transition metal complexes tend to have poorer solubility in low-polarity media as compared to purely organic materials, which can be problematic for solution processing.^11^

The heavy atom effect (HAE) in nonmetallic compounds has previously been reported in naphthalimide and fluorene derivatives by using halogens or chalcogen atoms to promote phosphorescence.^12−14^ Moreover, highly efficient and persistent room-temperature phosphorescence (RTP) can be obtained by combining the HAE and halogen bonding.^15,16^ For example, functionalization of 9-(4-(phenylsulfonyl)benzyl)-9*H*-carbazole with bromine (CzS2Br) leads to a phosphorescence quantum yield (Φ_p_) of 52% and a long photoluminescence lifetime of 152 ms because of intramolecular halogen bonding that restrains the molecular motion and reduces nonradiative decay.^17^ In dioxophenothiazine derivatives, phosphorescence is promoted through intermolecular halogen bonding, and the Φ_p_ increases significantly from 0.5% to 11.1%, with a phosphorescence lifetime of 391 ms.^18^

The enhancement of SOC through the HAE can also be useful for increasing the reverse intersystem crossing (RISC) rate in TADF compounds.^19^ For example, functionalization of 2,4,5,6-tetrakis(9*H*-carbazol-9-yl)isophthalonitrile (4CzIPN) with iodine has been reported to result in a decrease of the delayed fluorescence lifetime and an increase of the RISC rate from 0.77 × 10^6^ s^–1^ to 1.5 × 10^7^ s^–1^.^20^ Baldo et al. also reported the proximity (external) HAE from halogenated derivatives of the 4,4′-bis(*N*-carbazolyl)-1,1′-biphenyl (CBP) host doped with 4CzIPN guest molecules.^21^

To better understand the impact of halogenation on the photophysical properties of a commonly used organic semiconductor host material, 1,3-bis(*N*-carbazolyl)benzene (mCP), we investigated the effect of the type and number of halogens on the ISC rate.^22−26^ In the first part of the paper, the kinetic rate constants are obtained through a combination of steady-state and time-resolved photoluminescence (PL) experiments, and the results are compared with those obtained from density functional theory (DFT) calculations. A better understanding of the relationship between the presence of halogen atoms and the photophysical properties of the resulting molecules is important for the design of novel compounds with enhanced phosphorescence and delayed fluorescence. Similarly to ref (21), in the second part of the paper, the use of these molecules as hosts for dopant molecules showing TADF is explored, by doping with the emitter 7,10-bis(4-(diphenylamino)phenyl)-2,3-dicyanopyrazino-phenanthrene (TPA-DCPP). A combination of transient PL and PL quantum yield measurements allows us to obtain the RISC rate, which is modulated via the external HAE. We find a 4-fold increase in RISC rate for the iodine-containing derivative. The use of the external HAE can be an attractive route for reducing the steady-state triplet population in known TADF emitters, which is problematic for both roll-off and stability at high current density.

## Experimental Methods

### Materials and General Experimental Information

All commercially available chemicals and reagent grade solvents were used as received. Air-sensitive reactions were performed using standard Schlenk techniques under a nitrogen atmosphere. Flash column chromatography was carried out using silica gel (60 Å, 40–63 μm). Analytical thin-layer chromatography (TLC) was performed using silica plates with aluminum backings (250 μm with F-254 indicator) and visualized using a 254/365 nm UV lamp. Solution ^1^H and ^13^C NMR spectra were recorded in CDCl_3_ on an NMR spectrometer (400 MHz for ^1^H and 101 MHz for ^13^C). The NMR signal is described as follows: s = singlet, d = doublet, t = triplet, dd = doublet of doublets, td = triplet of doublets, ddd = doublet of doublets of doublets, and m = multiplets. Melting points were measured using open-ended capillaries on an Electrothermal Mel-Temp melting point apparatus and are uncorrected. High-resolution mass spectrometry (HRMS) analyses were performed at the BSRC Mass Spectrometry and Proteomics Facility, University of St Andrews, using a ThermoScientific LCQ Fleet Ion Trap Mass Spectrometer equipped with Ultimate 3000 LC and National Mass Spectrometry Facility at Swansea using a Waters Xevo G2-S QTof mass spectrometer. HPLC traces were obtained using an ACE Excel 2 C18 analytical (3 × 150 mm) column.

### Sample Preparation

The mCP derivatives were purified by thermal gradient sublimation. Thermogravimetric analysis (TGA 8000, PerkinElmer) was performed to avoid exceeding the decomposition temperature. The powder was placed in a crucible in a quartz tube under high vacuum (∼10^–6^ Torr) and positioned in a gradient temperature furnace (Zhengzhou Protech Technology Co., Ltd. PT-1200T). The powder, located in the hottest zone of the furnace, evaporated and condensed in the tube at a lower temperature.

Thin-film fabrication was performed by spin coating under N_2_ on 1 cm^2^ silicon substrates (Wafer Pro) using a concentration of 1 wt % mCP (Luminescence Technology Corp.) in PMMA (Sigma-Aldrich, 120000 g·mol^–1^) or 10 wt % TPA-DCPP in halogen mCP hosts. The solution was diluted to a concentration of 5 wt % in anhydrous toluene (Sigma-Aldrich), stirred overnight, and then filtered using PTFE filters (Cole-Parmer Essentials 0.2 μm Pore). The spin-coating speed was set at 2500 rpm for 45 s. The samples were then dried at 30 °C for 20 min.

### Steady-State and Transient PL and Absorption

The samples were cooled to 6 K using the Advanced Research Systems DE-204S cryostat, and the temperature was controlled using the Cryo-Con 22C temperature controller. The mCP was excited at 325 nm using a femtosecond Yb:KGW laser (Light Conversion PHAROS Femtosecond Laser) coupled with an optical parametric amplifier (ORPHEUS-F) and second-harmonic generator (LYRA-SH). The output of the second-harmonic generator was filtered using a 420 nm short-pass filter. Photoluminescence spectra were collected using a CCD camera (Pixis 400BExcelon) coupled to a monochromator with a 15 μm slit opening. Nanosecond transient photoluminescence spectra were acquired using a streak camera (Hamamatsu Photonics High Dynamic Range Streak Camera C7700), and the delay was adjusted using a delay generator (Stanford Research Systems DG645). Transient photoluminescence spectra at a longer time range were acquired using a GoPro Hero11 camera at 240 fps. Absorption spectra were obtained by diluting the compounds in anhydrous toluene under N_2_ at a concentration of 10^–5^ mol·L^–1^ using a spectrophotometer (PerkinElmer Lambda 25 UV/vis) and quartz cuvettes (Hellma X2) with a 1 cm optical path.

### Computational Studies

The computations were performed using the Compute Canada resources and facilities. Molecular structures were visualized using GaussView software. Calculations were performed using Gaussian 16. Relativistic calculations and SOCME were carried out using ORCA 5.0.1 software.^27^ The basis sets used were obtained from the Basis Set Exchange platform.^28^ The solvation effect was modeled using the polarizable continuum model (PCM).^29^ The 6-31+g(d,p) basis set was chosen following a convergence calculation of electronic energy and molecular geometry. Twenty conformers were generated as initial structures for geometry optimization, and a frequency analysis was performed and revealed no imaginary frequencies, indicating convergence to an energy minimum.

## Results

Five halogen-substituted mCP derivatives were synthesized to study the impact of halogenation on the photophysical properties (Figure 1). The mCP derivatives with different halogens (mCP-Br and mCP-I) placed on the central phenyl ring were used to investigate the influence of the halogen type. To study the impact of the number and position of the halogens, additional bromine atoms were attached to the carbazole units of mCP-Br_2_, mCP-Br_3_, and mCP-Br_5_. The synthesis procedure and NMR are provided in the Supporting Information (Figures S1–S20).

**Figure 1:**
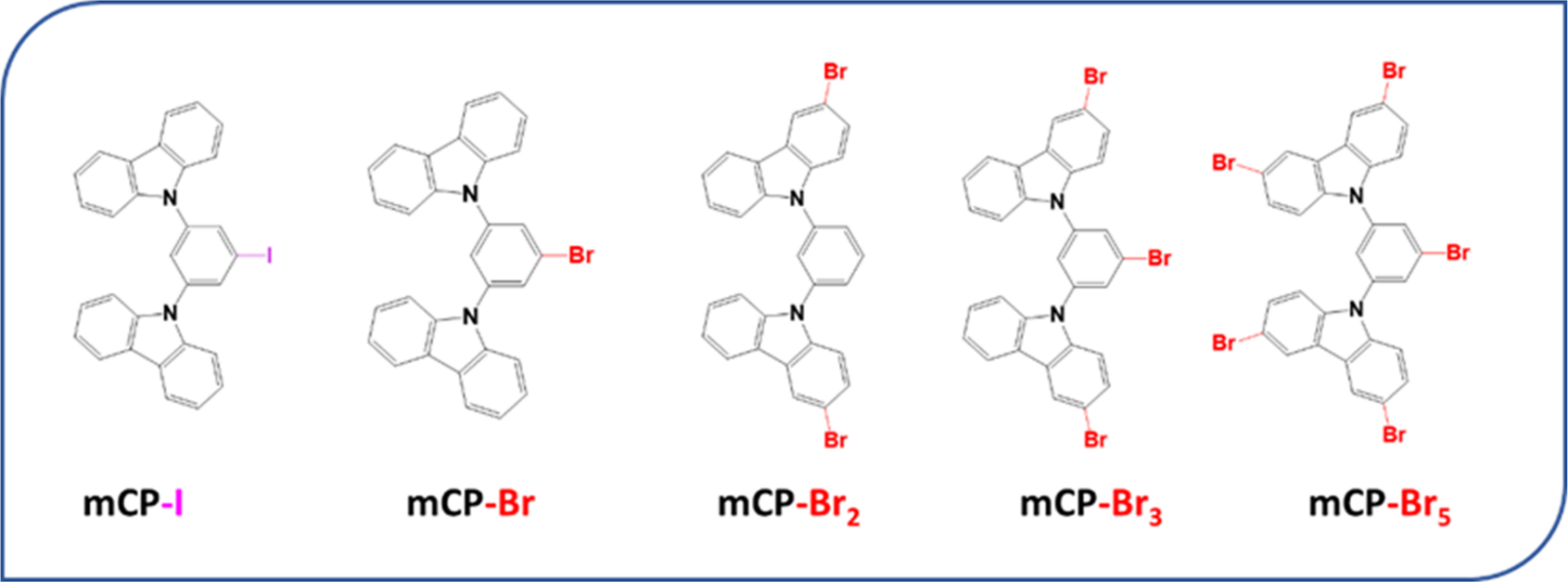
Molecular structure of the different mCP derivatives.

Thin films of mCP and of its derivatives doped in a poly(methyl methacrylate) (PMMA) matrix were prepared by spin-coating at a concentration of 1 wt % to minimize intermolecular interactions and energy transfer between dopant molecules. All of the data presented in the main text were taken at 6 K to minimize nonradiative decay and allow for efficient phosphorescence. Temperature-dependent PL can be found in the Supporting Information (Figure S21).

**Figure 2:**
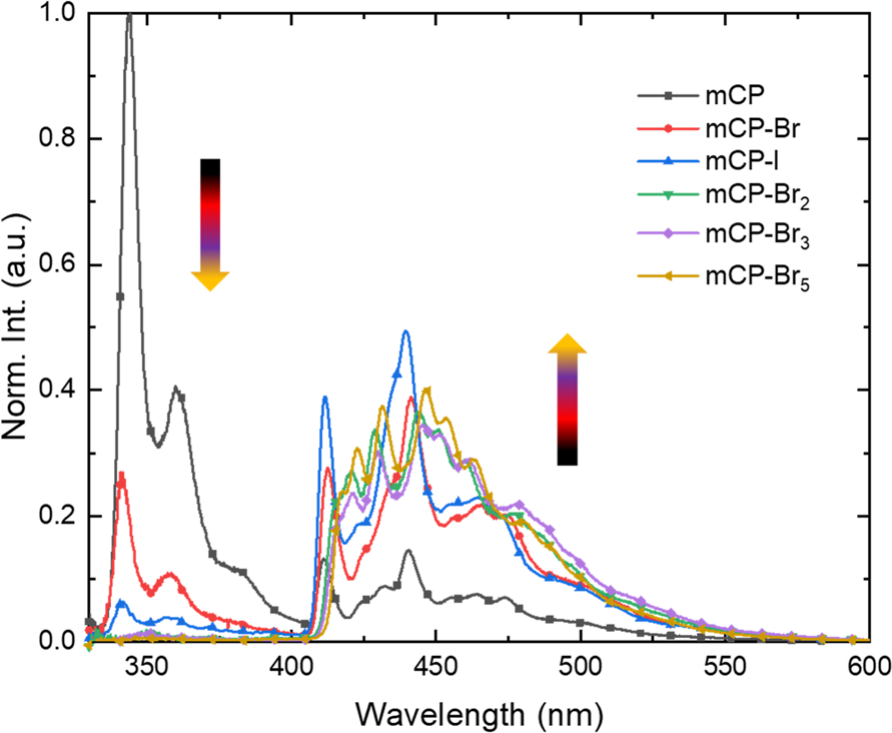
Photoluminescence at 6 K, which shows fluorescence (330–400 nm) and phosphorescence (400–600 nm) emission of mCP and its derivatives, excited at 320 nm. The spectra are normalized with respect to the total number of emitted photons.

The effect of halogenation on the 6 K PL is shown in Figure 2. We found that the addition of the halogens increases phosphorescence efficiency as compared to mCP, which occurs in the 400–600 nm spectral range, while simultaneously decreasing the fluorescence intensity, which occurs below 400 nm. The effect is more pronounced for mCP-I than mCP-Br and gradually increases with increasing number of bromine atoms. The relative fluorescence and phosphorescence quantum yields were calculated from the PL spectra by integrating under the curve to obtain the number of photons emitted within each spectral region. From mCP to mCP-Br_5_, we found a significant decrease of the fluorescence quantum yield (Φ_f_) from 73% to 0.8%, along with an increase of the low-temperature phosphorescence quantum yield (Φ_p_) from 27% to 99.2%.

As can be seen in Figure 2, the position and number of halogens affect the vibronic features in the phosphorescence spectra, but the fluorescence shape remains relatively unchanged. The introduction of halogens on the carbazole in mCP-Br_2_, mCP-Br_3_ and mCP-Br_5_ results in a redshift of 5 nm of the emission and an increase of the intensity of vibronic peaks at 423, 431, 454, and 463 nm as compared to mCP, while the presence of the halogen on the central phenyl moiety in mCP-Br and mCP-I did not change the shape of the phosphorescence spectra in these two compounds. The relative intensity of the two vibronic peaks at 410 and 441 nm in mCP is unaffected by the halogenation. The change in the vibronic structure of the triplet emission when bromine is bonded to the carbazole suggests a modification of the vibrational wave functions of the triplet state, which subsequently impacts the Franck–Condon factors and the intensity of the vibronic transitions.

**Figure 3:**
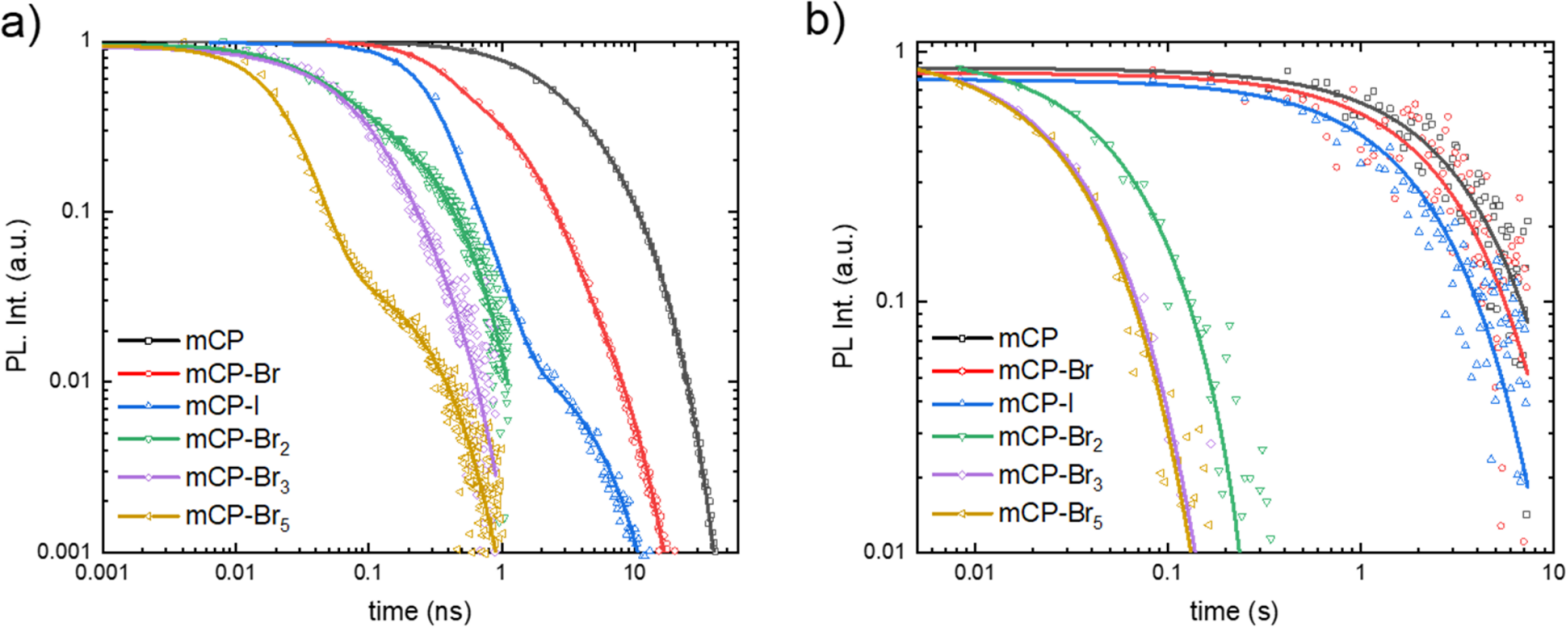
Transient (a) fluorescence (measured at 360 nm) and (b) phosphorescence (measured at 450 nm) of 1 wt % mCP derivatives doped in PMMA at 6 K (λ_exc_ = 320 nm). Note the widely different time scales and the effect of halogenation. Solid lines are (a) biexponential and (b) monoexponential fits to the data.

Figure 3 shows the time-resolved fluorescence (Figure 3a) and phosphorescence (Figure 3b) traces of the family of mCP derivatives. Figure 3a shows that the fluorescence lifetime is dramatically reduced by the presence of halogens in the mCP derivatives, down to values <100 ps. Solid lines show fits to a biexponential decay model with weighted average lifetimes (τ_f_) summarized in Table S1. This biexponential decay is attributed to a combination of aggregation and Förster resonance energy transfer (FRET). We have verified that monoexponential decay is recovered in drop cast films of 0.1 wt % in PMMA (Figure S22) at the cost of a significant reduction in signal. This interpretation was also provided in a previous work comparing transient absorption measurements of mCP in solution and in PMMA thin films.^22^ We have further verified that the trends in fluorescence and phosphorescence intensities across different mCP derivatives are not predominantly due to aggregation (Figure S23). In our 1 wt % films, we find that τ_f_ decreases from 5.7 ± 0.1 ns in mCP to 67 ± 0.5 ps in mCP-Br_5_. This reduction is notably more pronounced for mCP-I than for mCP-Br, with respective values of 1.04 ± 0.07 ns and 1.88 ± 0.05 ns. From Figure 3b, we found a similar trend for the weighted phosphorescence lifetime (τ_ph_) obtained from monoexponential fitting. The value of τ_ph_ decreases by 2 orders of magnitude from 3.1 ± 0.1 s for mCP to 28 ± 0.1 ms for mCP-Br_5_.

**Figure 4:**
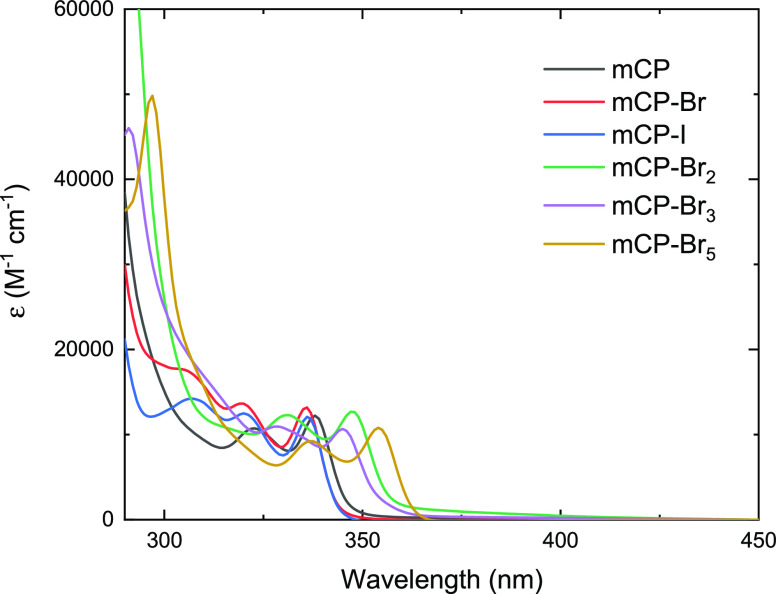
Absorption spectra of mCP and its halogenated derivatives in toluene at concentrations of 10^–5^ M.

Figure 4 shows the absorption spectra of the compounds in toluene. We found very similar molar extinction coefficients (ε) for the S_0_ → S_1_ transition across all of the compounds. However, the presence of the halogens shifts the absorption onset from 330 to 360 nm. The same effect can also be observed in the emission spectra, and the origin of this shift is explored below.

Based on these results, we can calculate the different decay rates at 6 K. Assuming that the nonradiative decay rate (*k*_nr_) is significantly smaller than the radiative decay rate (*k*_r_) and the ISC rate (*k*_ISC_) at 6 K, the decay rates are given by *k*_rS_ = Φ_f_τ_f_^–1^, *k*_ISC_ = Φ_ph_τ_f_^–1^, and *k*_rT_ = τ_ph_^–1^. These assumptions are supported by the temperature-dependent PL behavior, which shows that both fluorescence and phosphorescence intensities increase with decreasing temperature until they reach a plateau at 40 K (Figure S21). We ascribe this plateau to a near suppression of nonradiative decay rates due to the low thermal energy available in the environment to drive nuclear motion. Moreover, no delayed fluorescence is observed at 6 K indicating that RISC does not contribute to the singlet population at this temperature. The results are consistent with the study of Wu et al.^30^ Importantly, the analysis also leads to only modest changes in *k*_rS_ across the molecules, which is consistent with the small changes observed in the absorption spectra. Table 1 summarizes the calculated rate constants. Importantly, we found that an increase in the number of halogens enhances *k*_ISC_ by more than a factor of 300. The ISC rate ranges from (5.3 ± 0.4) × 10^7^ s^–1^ for mCP to (1.5 ± 0.1) × 10^10^ s^–1^ for mCP-Br_5_. The increase in *k*_ISC_ is three times greater for mCP-I than for the mCP-Br, which is consistent with a stronger SOC induced by the heavier iodine atom. The radiative decay rate from the triplet state also increases by a factor of 100, reaching a value of (36 ± 1) s^–1^ for mCP-Br_5_. In contrast, the radiative decay rate of the singlet remains relatively unchanged across all of the compounds.

**Table 1:** Summary of the Photophysical Properties of the mCP Derivatives at 6 K^a^

	τ_f_ (ns)	τ_ph_ (s)	Φ_f_ (%)	Φ_ph_ (%)	*k*_rS_ (10^8^ s^–1^)	*k*_rT_ (s^–1^)	*k*_ISC_ (10^8^ s^–1^)
mCP	5.7 ± 0.1	3.1 ± 0.1	70 ± 2	30 ± 2	1.23 ± 0.04	0.32 ± 0.01	0.53 ± 0.04
mCP-Br	1.88 ± 0.05	2.7 ± 0.1	19 ± 1	81 ± 1	1.01 ± 0.06	0.37 ± 0.01	4.3 ± 0.1
mCP-I	1.04 ± 0.07	1.96 ± 0.04	8.5 ± 0.4	91.5 ± 0.4	0.82 ± 0.07	0.51 ± 0.01	8.8 ± 0.6
mCP-Br_2_	0.222 ± 0.007	0.056 ± 0.003	1.8 ± 0.1	98.2 ± 0.1	0.81 ± 0.05	18 ± 1	44 ± 1
mCP-Br_3_	0.128 ± 0.01	0.030 ± 0.002	1.5 ± 0.1	98.5 ± 0.1	1.2 ± 0.1	31 ± 2	76 ± 6
mCP-Br_5_	0.067 ± 0.005	0.028 ± 0.001	0.8 ± 0.1	99.2 ± 0.1	1.2 ± 0.2	36 ± 1	150 ± 10

a τ_f_ and τ_ph_ are the fluorescence and phosphorescence lifetimes; Φ_f_ and Φ_ph_ are the fluorescence and phosphorescence quantum yields; and *k*_rS_, *k*_rT_, and *k*_ISC_ are the singlet, triplet, and intersystem crossing decay rate constants.

To gain insight into the basis of the relationship between halogen content of mCP and the optical properties of these derivatives, DFT and TD-DFT calculations were performed using each of the B3LYP, CAM-B3LYP, and LC-wPBE functionals. Compared to B3LYP, the use of CAM-B3LYP and LC-wPBE add a long-range correction term, essential to model charge transfer states and noncovalent interactions such as halogen bonds.^31,32^ The LANL2DZ effective core potential was used to model the heavy halogen atoms in the molecule, while the 6-31+g(d,p) basis set was used for the other atoms in the molecules.^33^

The optimized S_0_ geometry is expectedly similar for all of the compounds. The dihedral angles between the carbazole moieties and the central phenyl vary by only 3°, and the bond lengths between the carbazole and the phenyl are similar (Table S3). The addition of halogen atoms has therefore only a very minor impact on the molecular geometry at S_0_. These results are consistent with previous DFT calculations conducted on molecules that contain carbazole moieties bonded to a phenyl ring.^34,35^ Furthermore, the choice of the functional showed no notable differences on the final geometry.

Using the optimized S_0_ geometry, TD-DFT calculations were performed to calculate the energy and the oscillator strength of the S_0_ → S_1_ transition. The results are shown in Table 2. The calculations reveal that the oscillator strengths and absorption wavelengths strongly depend on the choice of functional. The dependence of the excited state energies on the choice of functional has already been reported for halogen analogues of 1,4-di(9*H*-carbazol-9-yl)benzene, but the most appropriate functional to use remained undetermined due to a lack of experimental data.^36^ The use of the B3LYP functional predicts a significant decrease of the oscillator strength for mCP-Br_2_ and mCP-Br_5_ compared to the other compounds, which is clearly inconsistent with experiment. Additionally, the variation of the calculated absorption wavelength for this functional does not follow the observed spectral shift (Table 2). In contrast, using the CAM-B3LYP and LC-wPBE functionals, the evolution of absorption wavelength and the small variation in oscillator strength between the different derivatives are in good agreement with the experimental absorption spectra and the modest changes in *k*_rS_. However, these functionals consistently show a 50 nm blueshift between the calculated and observed absorption spectrum. The long-range correction is essential to correctly model the shift in excitation energy and to obtain the correct oscillator strengths for these halogenated derivatives. However, the use of the long-range separated functionals also fails to accurately calculate the energy splitting between the singlet and the triplet states (Δ*E*_ST_) of mCP. The Δ*E*_ST_ is highly overestimated with CAM-B3LYP and is 1.25 eV for mCP. The use of the B3LYP functional leads to a more accurate value of 0.74 eV compared to the experimental one of 0.62 eV (Figure 2). It is well established that the proper tuning parameters in long-range corrected (LC) or Coulomb attenuated method (CAM) functionals is crucial to accurately obtain the energy levels of singlet and triplet states.^37−39^ For example, the tuned CAM-B3LYP (α = 0.10, β = 0.90, ω = 0.15) functional predicts a Δ*E*_ST_ of 0.70 eV, an absorption wavelength of 307 nm, and an accurate description of the spectral shift induced by halogen substitution. The evolution of the excitation energy and the Δ*E*_ST_ with tuning parameters is reported in the Supporting Information (Figure S24).

**Table 2:** Influence of the Functional for TD-DFT Calculation on the Absorption Wavelength and the Oscillator Strength (*f*) of the S_0_ → S_1_ Transition for the mCP Derivatives^a^

6-31+g(d,p)		mCP	mCP-Br	mCP-Br_2_	mCP-Br_3_	mCP-Br_5_	mCP-I
B3LYP	λ_abs_ (nm)	317	329	324	330	330	327
	*f*	0.005	0.1704	0.072	0.159	0.0057	0.161
LC-wPBE	λ_abs_ (nm)	270	269	274	273	276	269
	*f*	0.159	0.175	0.155	0.144	0.148	0.172
CAM-B3LYP	λ_abs_ (nm)	287	285	292	290	295	285
	*f*	0.140	0.167	0.137	0.129	0.132	0.161
Tuned CAM-B3LYP	λ_abs_ (nm)	307	304	314	312	319	305
	*f*	0.104	0.123	0.104	0.087	0.097	0.11
Experimental	λ_abs_ (nm)	338	336	347	346	354	337
	ε (M^–1^ cm^–1^)	12230	13251	12680	10443	10797	12079

a Experimental absorption wavelength (λ_abs_) and extinction molar coefficient (ε) values are included for comparison.

**Figure 5:**
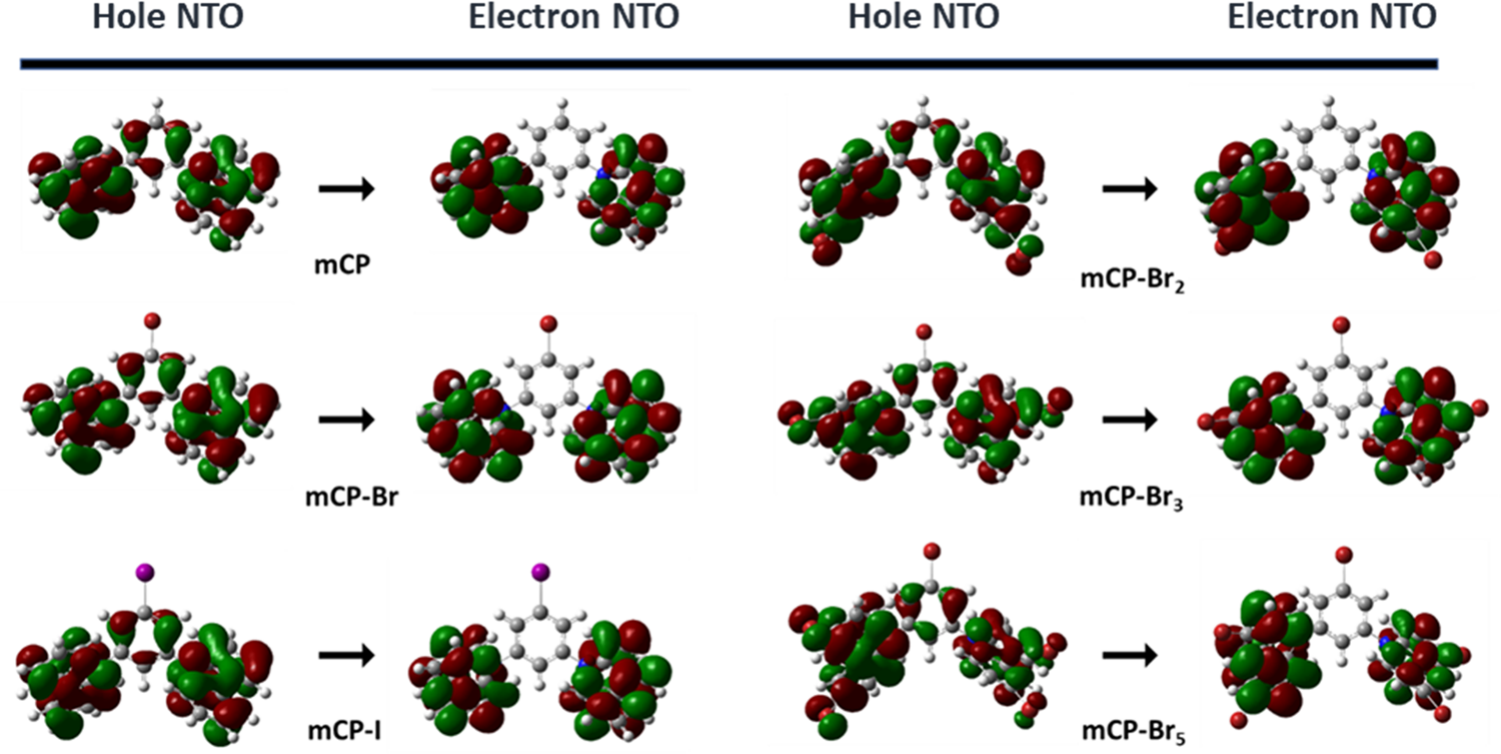
Natural transition orbitals (NTOs) of S_0_ → S_1_ at the tuned CAM-B3LYP/6-31+g(d,p) level (ISO = 0.02).

To understand the spectral shifts, we examined the natural transition orbitals (NTOs) obtained from TD-DFT calculations. Figure 5 shows that the electron NTO is delocalized only on the carbazole units, while the hole NTO has some additional delocalization on the central phenyl ring. We find that functionalization with halogens on the carbazole contributes to the delocalization of the hole NTO. The lone pairs of electrons of bromine atoms contribute to the resonance system and act as an electron-donating group. This effect increases the electronic density in the hole NTO and raises the overall energy of the orbital. It results in a reduction in the energy gap between the transition orbitals and a red-shift in the absorption and emission spectra. This is consistent with the observed trends in the absorption and emission spectra, where the addition of halogens to the phenyl results in a very small difference in each of the absorption and emission wavelengths, whereas their addition on the carbazole leads to a more pronounced red-shift.

To understand the influence of the type and number of halogen on the SOC, relativistic calculations were performed using the Zero-Order Regular Approximation (ZORA) method.^40^ The spin–orbit coupling matrix elements (SOCMEs) are highly dependent on the energy level splitting between the triplet and singlet states. For this reason, they were carried out at the B3LYP/ZORA-TZVP level. Table 3 collates the results of the calculation of the SOCME between the S_1_ state and the lower energy triplet states T_*n*_ (*n* = 1–8) at the S_1_ optimized geometry. The results show an increase in the SOCME for all halogenated compounds as compared to mCP.

**Table 3:** Comparison of SOCME Values of the mCP Derivatives between S_1_ and T_*n*_ (*n* = 1–8) from B3LYP/ZORA-TZVP Calculations

|⟨T_*i*_|*H*_SO_|S_*j*_⟩| (cm^–1^)	mCP	mCP-Br	mCP-I	mCP-Br_2_	mCP-Br_3_	mCP-Br_5_
|⟨T_1_|*H*_SO_|S_1_⟩|	0.20	0.10	0.13	0.34	0.00	1.06
|⟨T_2_|*H*_SO_|S_1_⟩|	0.22	0.20	0.15	0.49	0.10	1.23
|⟨T_3_|*H*_SO_|S_1_⟩|	0.52	3.00	9.84	0.06	2.66	2.35
|⟨T_4_|*H*_SO_|S_1_⟩|	0.00	0.17	0.35	0.13	0.00	0.10
|⟨T_5_|*H*_SO_|S_1_⟩|	0.59	1.90	5.76	1.20	3.98	4.49
|⟨T_6_|*H*_SO_|S_1_⟩|	0.17	2.08	6.04	0.06	2.01	2.04
|⟨T_7_|*H*_SO_|S_1_⟩|	0.28	1.27	4.91	0.83	2.14	1.30
|⟨T_8_|*H*_SO_|S_1_⟩|	0.00	0.58	1.07	0.71	0.17	0.14

The increase is particularly significant for coupling between S_1_ to each of T_3_, T_5_, T_6_, and T_7_ states. The SOCME between S_1_ and T_5_ changes by an order of magnitude from 0.59 cm^–1^ for mCP to 4.49 cm^–1^ for mCP-Br_5_. The effect is increased with the heavier iodine atom with a SOCME of 5.76 cm^–1^ between S_1_ and T_5_ and 9.84 cm^–1^ between S_1_ and T_3_, which is consistent with the faster ISC rates that we observe experimentally for mCP-I. We also find that both the position and number of the halogen have a significant effect on the SOCME with the different triplet states. The calculations reveal that the increase in SOCME is smaller when bromine functionalization is only present on the carbazole units (e.g., mCP-Br_2_) as compared to the phenyl (e.g., mCP-Br) despite the presence of two bromines on the molecule. Relativistic calculations conducted on similar molecules (PDCz) have already shown that the position of bromine on carbazole units can have a significant effect on the SOCME.^36^ One can link the SOCME to *k*_ISC_ by recalling that ISC can be expressed as a sum of the SOCMEs weighted by the vibrational overlaps between S_1_ and the different triplet states.^5^ Given that we find a large experimental increase in the *k*_ISC_ when going from mCP-Br to mCP-Br_2_, it is likely that the change in vibrational overlap plays an important role in dictating the *k*_ISC_ in these compounds in addition to the SOCMEs. Indeed, this would be consistent with the stark change in the intensity of the phosphorescence vibronic peaks observed between mCP/mCP-Br and mCP-Br_2/3/5_, where for the latter bromine is present on the carbazole unit.

**Figure 6:**
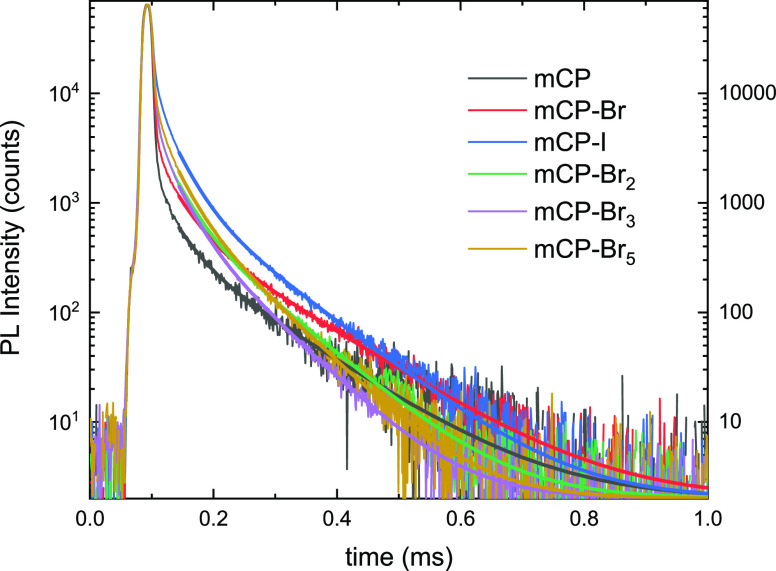
Transient PL of doped thin films of 10 wt % TPA-DCPP in different halogen mCP hosts (λ_exc_ = 600 nm; λ_em_ = 630 nm). Fits correspond to delayed fluorescence part of the emission.

To study the external heavy atom effect induced by the halogenation of the mCP, we prepared thin films of 10 wt % TPA-DCPP, a red TADF molecule,^41^ doped in the different mCP analogues. A combination of transient PL and PL quantum yield measurements was used to measure the RISC rate of the dopant molecule. Figure 6 shows the delayed fluorescence traces for TPA-DCPP in the different host molecules. The decay is multiexponential, which is consistent with a disorder-broadened distribution of RISC activation energies. The reported delayed fluorescence lifetimes, τ_d_, were obtained from a biexponential fit (Table S2). We found that τ_d_ was shorter in the halogenated hosts and decreases from 40 ± 3 μs for mCP to 30.8 ± 0.5 μs for mCP-Br_5_. The nature of the halogen also has a significant effect. We found a τ_d_ of 41 ± 1 μs for mCP-Br, which is similar to mCP, whereas we find 33.7 ± 0.3 μs for mCP-I (Table 4).

**Table 4:** Summary of Photophysical Properties and Calculated RISC Rate of Doped Thin Films of 10 wt % of TPA-DCPP in Different mCP Analogues^a^

Host	τ_d_ (μs)	Φ (%)	Φ_p_ (%)	Φ_d_ (%)	*k*_RISC_ (10^3^ s^–1^)
mCP	40 ± 3	59 ± 3	51 ± 3	8.1 ± 0.5	8.1 ± 0.8
mCP-Br	41 ± 1	51 ± 3	40 ± 3	11.4 ± 0.7	11.7 ± 0.6
mCP-I	33.7 ± 0.3	46 ± 2	25 ± 2	21 ± 1	33 ± 1
mCP-Br_2_	34.2 ± 0.4	48 ± 2	35 ± 2	13.5 ± 0.7	17.4 ± 0.8
mCP-Br_3_	31.3 ± 0.4	51 ± 3	36 ± 3	15 ± 1	20 ± 1
mCP-Br_5_	30.8 ± 0.5	53 ± 3	34 ± 3	19 ± 1	27 ± 1

a τ_d_ is the delayed fluorescence lifetime; Φ, Φ_p_, and Φ_d_ are the total, prompt, and delayed fluorescence quantum yields; and *k*_RISC_ is the reverse intersystem crossing decay rate.

The host matrix plays a crucial role in the photophysics of TADF emitters by affecting different excited states through dipole–dipole interactions.^26^ The properties of TPA-DCPP are known to be highly dependent on the host and the doping concentration because of aggregation concentration quenching, rigidity, and polarity effects.^42^ The halogenation of the hosts induced a relatively small variation of the PL quantum yield, Φ, from 59 ± 3% to 46 ± 2%, which does not compromise their use as a red TADF emitter or sensitizer.^43^ Moreover, the absorption and emission spectra of TPA-DCPP remain nearly unchanged when halogenated hosts are used instead of mCP (Figure S25). The delayed (Φ_d_) and prompt (Φ_p_) fluorescence quantum yield were estimated from the proportion of prompt and delayed fluorescence in the transient PL. By fitting the areas under the curve, we found that Φ_d_ of TPA-DCPP increases from 8.1 ± 0.5%, when doped in mCP, to 21 ± 1%for mCP-I and 19 ± 1% for mCP-Br_5_.

The RISC rate was estimated from delayed fluorescence lifetime and prompt and delayed quantum yield using^44^
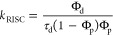
This commonly used expression assumes that singlet nonradiative decay is solely due to ISC. While this has not been explicitly confirmed, we can rule out nonradiative decay from concentration quenching given that the PLQY of TPA-DCPP has been shown to remain unchanged at doping concentrations ranging from 3 to 10 wt %.^45^

The presence of heavy atoms on the hosts resulted in an increase of the TADF dopant RISC rate. The RISC rate using mCP as host is (8.1 ± 0.8) × 10^3^ s^–1^, and the increase is particularly significant for mCP-Br_5_ and mCP-I with respective values of (2.7 ± 0.1) × 10^4^ s^–1^ and (3.3 ± 0.1) × 10^4^ s^–1^. This highlights that both the number and type of halogens on the host have an influence on the dopant RISC rate. A similar experiment was performed with 1,2,3,5-tetrakis(carbazol-9-yl)-4,6-dicyanobenzene (4CzIPN) (Figure S26), and the results are reported in Table S4.^2^ The RISC rate of 4CzIPN of this TADF molecule is a hundred times greater than TPA-DCPP in mCP, but it showed a similar 4-fold increase of the RISC rate from (1.18 ± 0.09) × 10^6^ s^–1^ in mCP to (4.0 ± 0.3) × 10^6^ s^–1^ in mCP-Br_5_ (Table S5). The increase is similar to that previously observed for 4CzIPN in brominated CBP.^21^ This underlines the versatility of this approach to enhance the RISC rate of various TADF dopants.

## Conclusion

In summary, we have shown the significant influence of halogenation on the intersystem crossing and phosphorescence rates of a family of halogenated mCP derivatives. The addition of halogens leads to an enhancement of *k*_ISC_ for all derivatives ranging from (5.3 ± 0.4) × 10^7^ s^–1^ for mCP to (1.5 ± 0.1) × 10^10^ s^–1^ for the mCP-Br_5_. In contrast, the absorption spectra and singlet radiative decay rates remain relatively unchanged. These observations are in agreement with TD-DFT calculations, but only when a long-range corrected functional is used such as CAM-B3LYP. However, the use of CAM-B3LYP with default parameters (μ = 0.33, α = 0.19, and β = 0.46) leads to a large overestimation of the energy difference between S_1_ and T_1_ and a large systematic shift between calculated and experimental absorption wavelengths. The use of the empirically tuned CAM-B3LYP (μ = 0.15, α = 0.10, and β = 0.90) functional reproduces well the spectral shift induced by the halogens, the trend in oscillator strengths, and the Δ*E*_ST_, while also minimizing the spectral shift in the absorption. The calculation of the SOCMEs combined with the phosphorescence spectra and measured rates also shows that the position of the halogen can have a large impact on both the magnitude of the SOC and the vibrational wave functions of the excited triplet states. Both need to be considered to accurately reproduce trends in ISC. We have also shown that the use of the halogen-functionalized mCP as a host molecule for the TADF dopant TPA-DCPP results in an increase of the RISC rate through the proximity heavy atom effect. The highest increase is obtained when mCP is functionalized with the heavier iodine atom or with five bromines, resulting in an approximately 4-fold increase in rate. In summary, we have shown how halogen functionalization of a commonly used OLED host material can be used to tune SOC and dramatically modify the photophysical behavior of the host molecule and increase the RISC rate of TADF dopant molecules through external SOC. This approach can be useful to increase intersystem crossing and phosphorescent yields in organic compounds, without the need for metal atoms, and to improve the stability and performance of OLEDs using existing TADF dopants.

## Supporting Information Available

Synthesis, spectra, temperature dependence PL, fit parameters, ground state geometry optimized by DFT, and 4CzIPN RISC rate calculation (PDF)
