# Supplementary material for: Heavy Atom Effect in Halogenated mCP and Its Influence on the Efficiency of the Thermally Activated Delayed Fluorescence of Dopant Molecules
Source: J Phys Chem C Nanomater Interfaces. 2024 Jan 17;128(3):1122–30. doi: 10.1021/acs.jpcc.3c05567 (PMC10823469; doi:10.1021/acs.jpcc.3c05567)
Supplement: Supplementary file 1 — jp3c05567_si_001.pdf [file jp3c05567_si_001.pdf]

Supporting Information

The heavy atom effect in halogenated mCP and its influence on the efficiency of the thermally activated delayed fluorescence of dopant molecules

Alexandre Malinge †, Shiv Kumar ◊, Dongyang Chen ◊, Eli Zysman-Colman ◊, Stéphane Kéna-Cohen* †*

† Department of Engineering Physics, École Polytechnique de Montréal, PO Box 6079, succ. Centre-Ville, Montreal QC H3C 3A7, Canada

◊ Organic Semiconductor Centre, EaStCHEM, School of Chemistry, University of St Andrews, St Andrews, Fife KY16 9ST, United Kingdom

Corresponding Author

E-mail: eli.zysman-colman@st-andrews.ac.uk

E-mail: s.kena-cohen@polymtl.ca

Table of Contents

Synthesis S3
General synthetic procedure S3
9,9'-(5-iodo-1,3-phenylene)bis(9*H*-carbazole) (mCP-I) S3
9,9'-(5-bromo-1,3-phenylene)bis(9*H*-carbazole) (mCP-Br) S4
1,3-bis(3-bromo-9*H*-carbazol-9-yl)benzene (mCP-Br_2) S4
9,9'-(5-bromo-1,3-phenylene)bis(3-bromo-9*H*-carbazole) (mCP-Br_3) S5
9,9'-(5-bromo-1,3-phenylene)bis(3,6-dibromo-9*H*-carbazole) (mCP-Br_5) S5
Spectra S6
mCP-I S6
mCP-Br S10
mCP-Br_2 .... S13
mCP-Br_3 .... S17
mCP-Br_5 .... S21
Temperature-dependent PL S25
Fit parameters S27
Ground state geometry optimization S29
TD-DFT calculation S30
RISC rate of 4CzIPN doped films S32

Synthesis

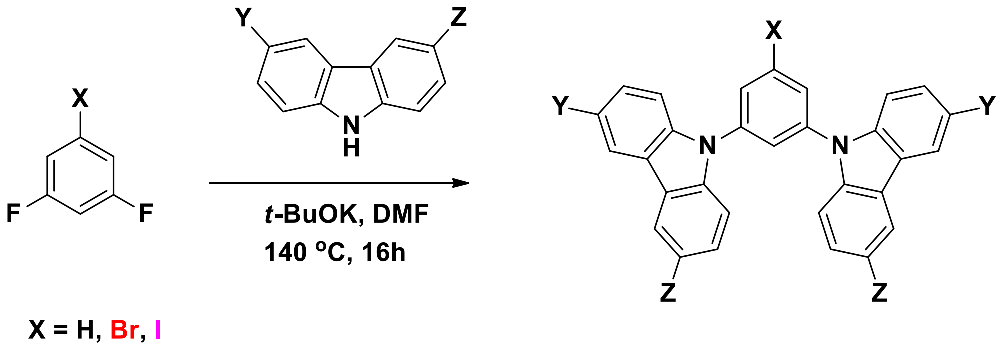

X= I, Y=H, Z=H, mCP-I
X=Br, Y=H, Z=H, mCP-Br
X=H, Y=Br, Z=H, mCP-Br_2
X=Br, Y=Br, Z=H, mCP-Br_3
X=Br, Y=Br, Z=Br, mCP-Br_5

General synthetic procedure

3-Bromocarbazole and 3,6-dibromocarbazole were prepared according to the literature.[1] A mixture of 3,5-difluorobromobenzene/3,5-difluoroiodobenzene (0.3 g, 1 equiv.), carbazole/3-bromocarbazole/3,6-dibromocarbazole (2.2 equiv.) and potassium *t*-butoxide (4.0 equiv.) in DMF (5 mL) was heated to 140 °C for 16 h. After cooling to room temperature, the reaction mixture was diluted with DCM (100 ml) and washed with water (3 × 50mL). The organic layer was dried over anhydrous sodium sulfate and the solvent was evaporated under reduced pressure. The crude product was purified by flash column chromatography on silica gel.

9,9'-(5-iodo-1,3-phenylene)bis(9*H*-carbazole) (mCP-I)

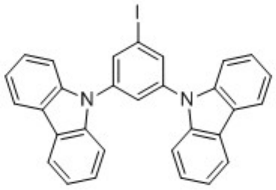

White solid. **Yield:** 0.51g, 30%. **R$_f$:** 0.5 (20% DCM/hexane). **Mp:** 196-198 °C (lit.[2] 193-194.5 °C). **^{1}H NMR (400 MHz, CDCl$_3$) δ**: 8.15 (d, *J* = 7.7 Hz, 4H), 8.06 (d, *J* = 1.9 Hz, 2H), 7.82 (t, *J* = 1.9 Hz, 1H), 7.54 (d, *J* = 8.2 Hz, 4H), 7.51 – 7.43 (m, 4H), 7.37 – 7.29 (m, 4H). **^{13}C{^{1}H} NMR (101 MHz, CDCl$_3$) δ**: 140.25, 134.47, 126.33, 124.69, 123.72, 120.70, 120.53, 109.56, 95.02. **HRMS (m/z):** 534.0593; **Found:** 534.0583. **HPLC**: 100% MeOH, 0.9 ml min^{-1}, 300 nm; tr (99.6%) = 2.33 min. Characterisation is in agreement with that reported in the literature.

9,9'-(5-bromo-1,3-phenylene)bis(9*H*-carbazole) (mCP-Br)

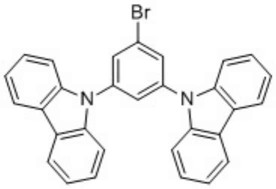

White solid. **Yield:** 1.01 g, 85%. **R$_f$:** 0.5 (5% EA/hexane). **Mp:** 226-228 °C (lit.[3] 211.8 °C). **^{1}H NMR (400 MHz, CDCl$_3$) δ:** 8.20 – 8.13 (m, 4H), 7.89 (d, *J* = 1.9 Hz, 2H), 7.82 (t, *J* = 1.9 Hz, 1H), 7.57 (dt, *J* = 8.2, 0.8 Hz, 4H), 7.52 – 7.45 (m, 4H), 7.38 – 7.32 (m, 4H). **^{13}C{^{1}H} NMR (101 MHz, CDCl$_3$) δ**: 140.44, 140.26, 128.62, 126.34, 124.09, 123.90, 123.77, 120.74, 120.54, 109.57. **HRMS (m/z):** 486.0732, 488.0711 **Found [M+H]$^+$:** 487.0807 (^{79}Br), 489. 0791 (^{81}Br). **HPLC**: 100% MeOH, 1ml min^{-1}, 300 nm; tr (97.3%) = 3.60 min. Characterisation is in agreement with that reported in the literature.

1,3-bis(3-bromo-9*H*-carbazol-9-yl)benzene (mCP-Br$_2$)

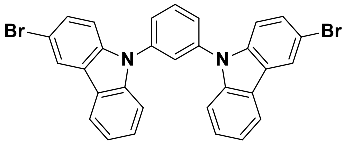

White solid. **Yield:** 1.02 g, 59%. **R$_f$:** 0.5 (10% EA/hexane). **Mp:** 213-217 °C (lit.[4] 119 °C). **^{1}H NMR (400 MHz, CDCl$_3$) δ:** 8.30 – 8.27 (m, 2H), 8.15 – 8.10 (m, 2H), 7.92 – 7.86 (m, 1H), 7.77

(t, J = 1.9 Hz, 1H), 7.71 (dd, J = 7.9, 2.0 Hz, 2H), 7.57 – 7.46 (m, 6H), 7.44 – 7.32 (m, 4H). **$^{13}C\{^{1}H\}$ NMR (101 MHz, $CDCl_3$) δ:** 140.90, 139.24, 139.04, 131.52, 128.88, 126.97, 126.08, 125.39, 125.16, 123.28, 122.55, 120.84, 120.71, 113.19, 111.12, 109.85. **HRMS (m/z):** 563.9831 **Found $[M]^+$:** 563.9806. **HPLC**: 100% MeOH, 1 ml min^{-1}, 300 nm; tr (95.4%) = 4.05 min. Characterisation is in agreement with that reported in the literature except for the melting point.

9,9'-(5-bromo-1,3-phenylene)bis(3-bromo-9*H*-carbazole) (mCP-Br_3)

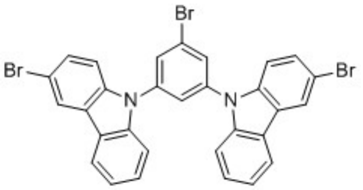

White solid. **Yield:** 1.23 g, 47% **R_f:** 0.5 (20% DCM/hexane). **Mp:** 213-217 °C**.** **^{1}H NMR (400 MHz, THF) δ:** 6.58 (d, *J* = 1.9 Hz, 2H), 6.53 (d, *J* = 1.8 Hz, 10H), 6.34 (dd, *J* = 9.0, 8.4 Hz, 13H), 6.26 – 6.16 (m, 12H), 6.12 (t, *J* = 1.6 Hz, 6H), 5.79 – 5.57 (m, 50H), 5.52 – 5.42 (m, 13H). **^{13}C NMR (101 MHz, THF) δ:** 138.99, 138.34, 137.28, 127.70, 127.24, 126.87, 125.07, 124.24, 123.77, 122.45, 122.24, 121.64, 121.20, 120.82, 119.04, 118.70, 118.29, 111.30, 109.75, 109.45, 107.94, 107.66. **HRMS (m/z):** 641.8936 **Found $[M]^+$:** 641.8938. **HPLC**: 100% MeCN, 0.7 ml min^{-1}, 300 nm; tr (99.7%) = 7.70 min.

9,9'-(5-bromo-1,3-phenylene)bis(3,6-dibromo-9*H*-carbazole) (mCP-Br_5)

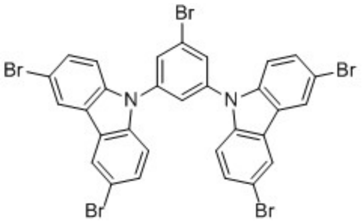

White solid. **Yield:** 1.13g, 50%. **R$_f$:** 0.5 (10% DCM/hexane). **Mp:** 210-214 °C. **^{1}H NMR (400 MHz, THF-*d*$_8$) δ:** 8.45 – 8.40 (m, 4H), 8.09 (d, *J* = 1.9 Hz, 2H), 7.97 (t, *J* = 1.9 Hz, 1H), 7.62 (dd, *J* = 8.8, 2.0 Hz, 4H), 7.56 – 7.51 (m, 4H). **^{13}C{^{1}H} NMR (101 MHz, THF-*d*$_8$) δ** 139.82, 139.55, 129.57, 129.42, 124.47, 124.36, 124.29, 123.52, 113.58, 111.57. **HRMS (m/z):** 797.7146 **Found [M]$^+$:** 797.7150. **HPLC**: 100% MeOH, 1 ml min^{-1}, 300 nm; tr (98.3%) = 4.48 min.

Spectra

mCP-I

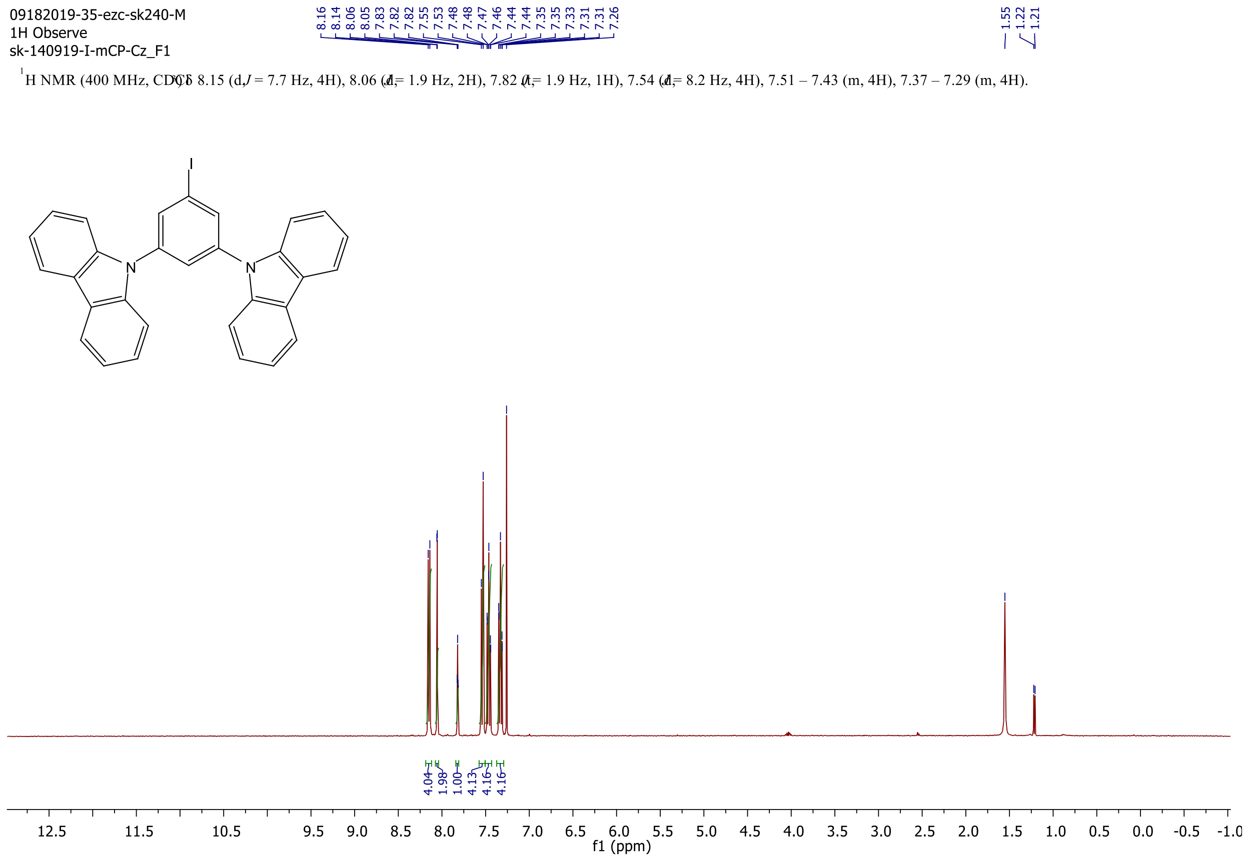

Figure **S1**. ^{1}H NMR of **mCP-I** in $CDCl_3$.

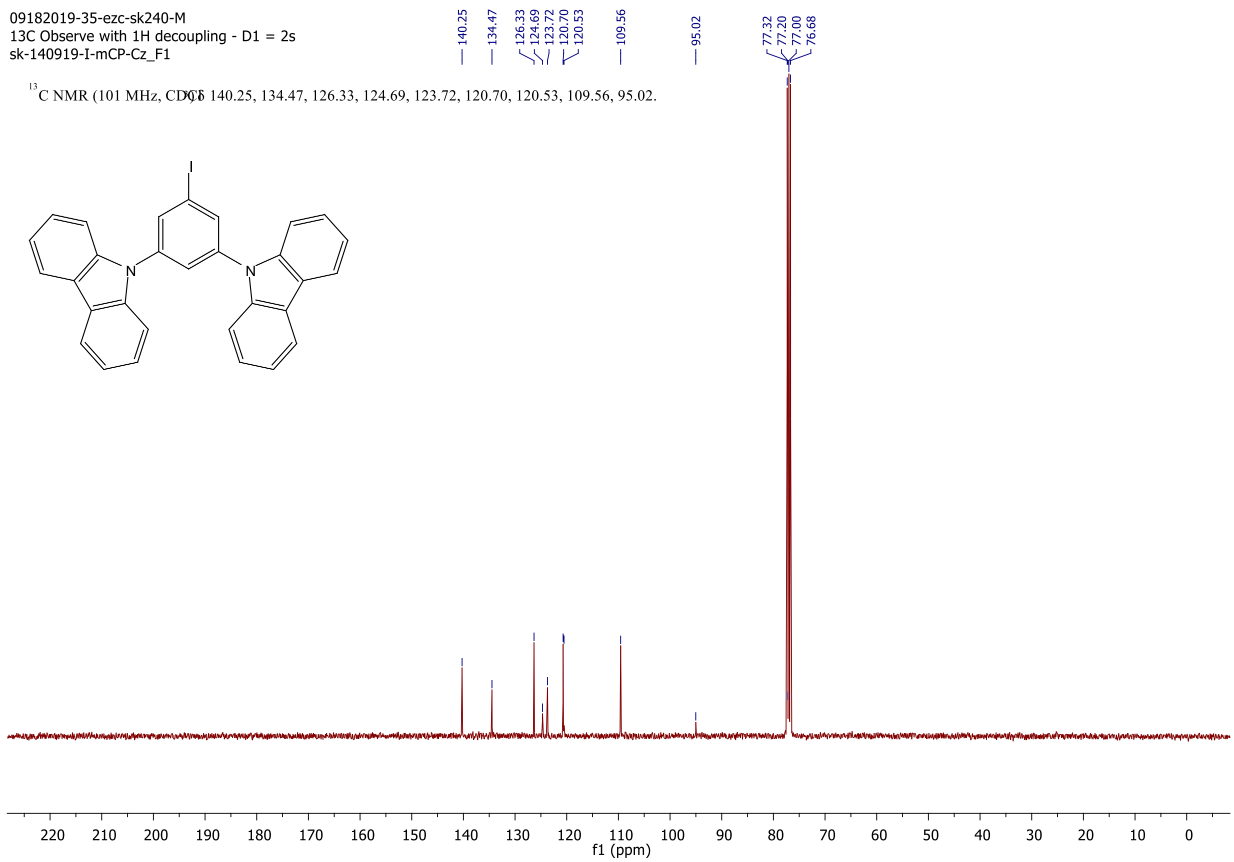

Figure **S2**. $^{13}C\{^{1}H\}$ NMR of **mCP-I** in $CDCl_3$.

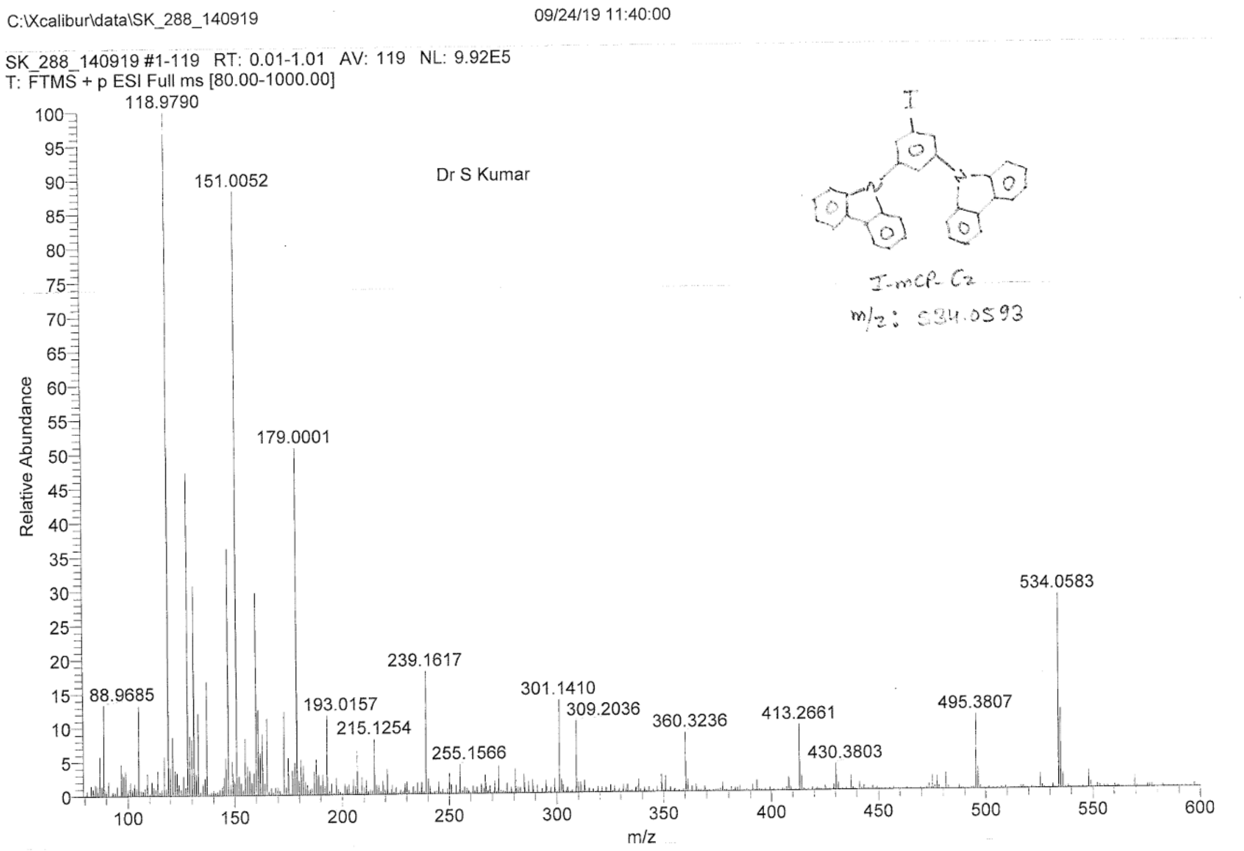

Figure **S3**. HRMS spectra of **mCP-I**.

HPLC Trace Report18Jun2023

<Sample Information>

Sample Name	: dc-ImCPICz		
Sample ID	:		
Method Filename	: 100% Methanol 20 mins-NEW.lcm		
Batch Filename	: dc-ImCPICz-MeOHnew		
Vial #	: 1-10	Sample Type	: Unknown
Injection Volume	: 60 uL		
Date Acquired	: 29/05/2023 17:31:40	Acquired by	: System Administrator
Date Processed	: 29/05/2023 17:51:42	Processed by	: System Administrator

<Chromatogram>

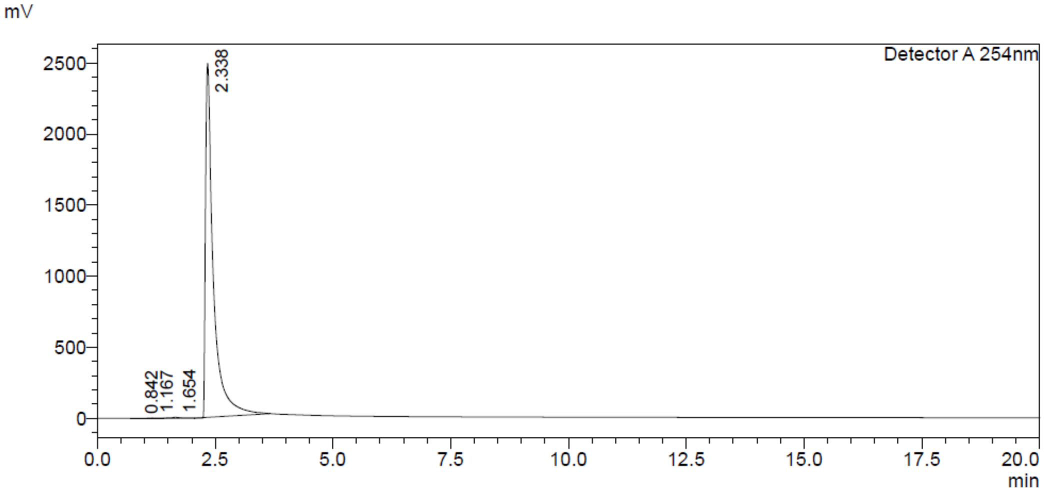

<Peak Table>

Detector A 254nm

Peak#	Ret. Time	Area	Height	Area%	Area/Height	Width at 5% Height
1	0.842	1250	122	0.004	10.207	--
2	1.167	25049	1967	0.086	12.735	--
3	1.654	77097	5770	0.264	13.362	--
4	2.338	29051152	2488748	99.645	11.673	0.520
Total		29154548	2496608	100.000		

Figure **S4**. HPLC trace of **mCP-I**.

mCP-Br

09052018-12-ezc-sk240-M
1H Observe
sk-II06-210818-F2

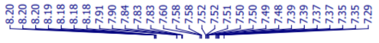

^{1}H NMR (400 MHz, $CDCl_3$) δ 8.23 – 8.15 (m, 4H), 7.90 (d, J = 1.9 Hz, 2H), 7.83 (t, J = 1.9 Hz, 1H), 7.62 – 7.56 (m, 4H), 7.50 (ddd, J = 8.3, 7.2, 1.2 Hz, 4H), 7.37 (td, J = 7.6, 1.0 Hz, 4H).

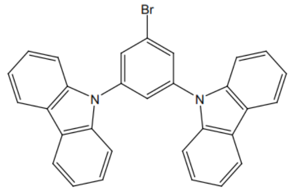

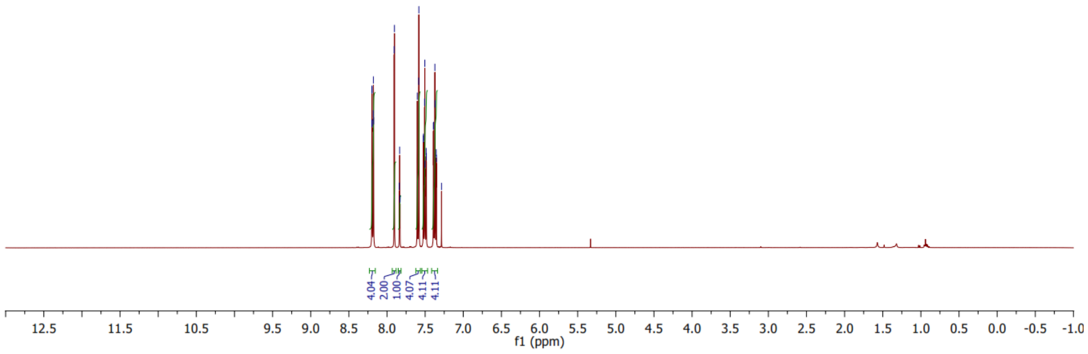

Figure **S5**. ^{1}H NMR of **mCP-Br** in $CDCl_3$**.**

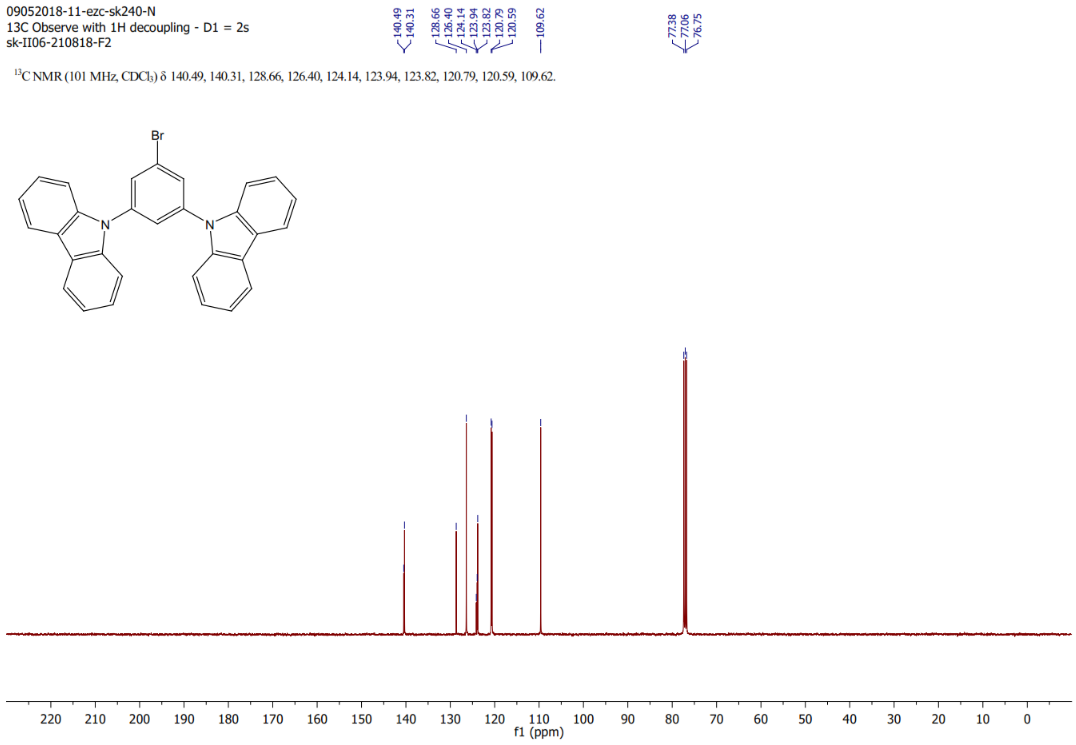

Figure **S6**. $^{13}C\{^{1}H\}$ NMR of **mCP-Br** in $CDCl_3$.

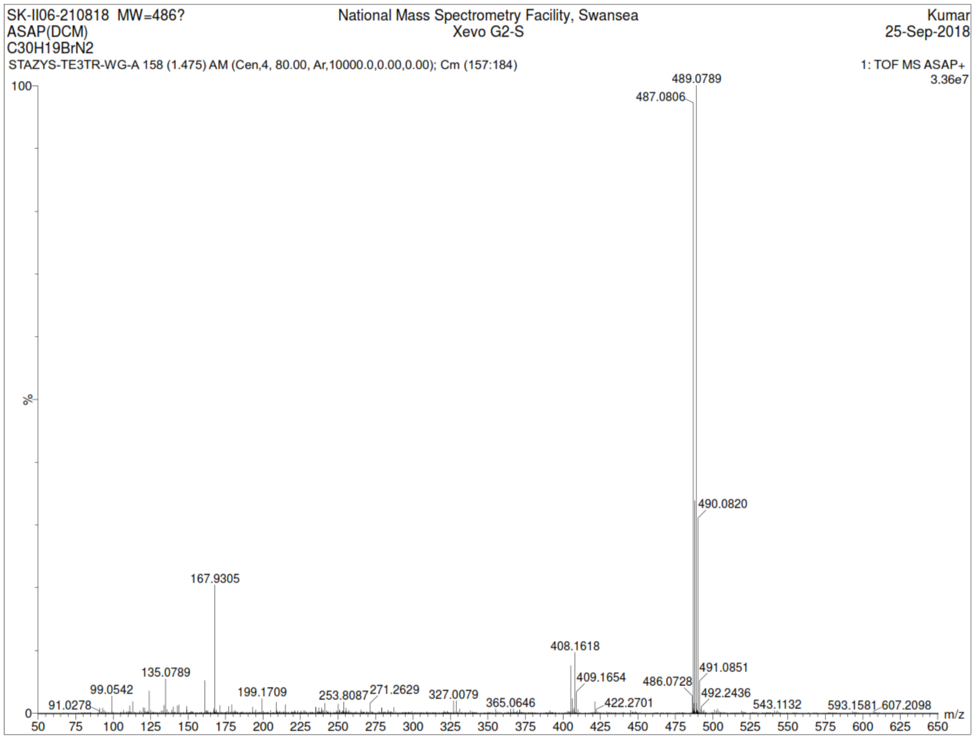

Figure **S7**. HRMS of **mCP-Br**.

HPLC Trace Report19Jun2023

<Sample Information>

Sample Name	: Br-mCP		
Sample ID	: SK-II06		
Method Filename	: 100% Methanol B 10 mins.lcm		
Batch Filename	: 05-03-2020.lcb		
Vial #	: 1-5	Sample Type	: Unknown
Injection Volume	: 5 uL		
Date Acquired	: 06/03/2020 17:25:44	Acquired by	: System Administrator
Date Processed	: 06/03/2020 17:45:55	Processed by	: System Administrator

<Chromatogram>

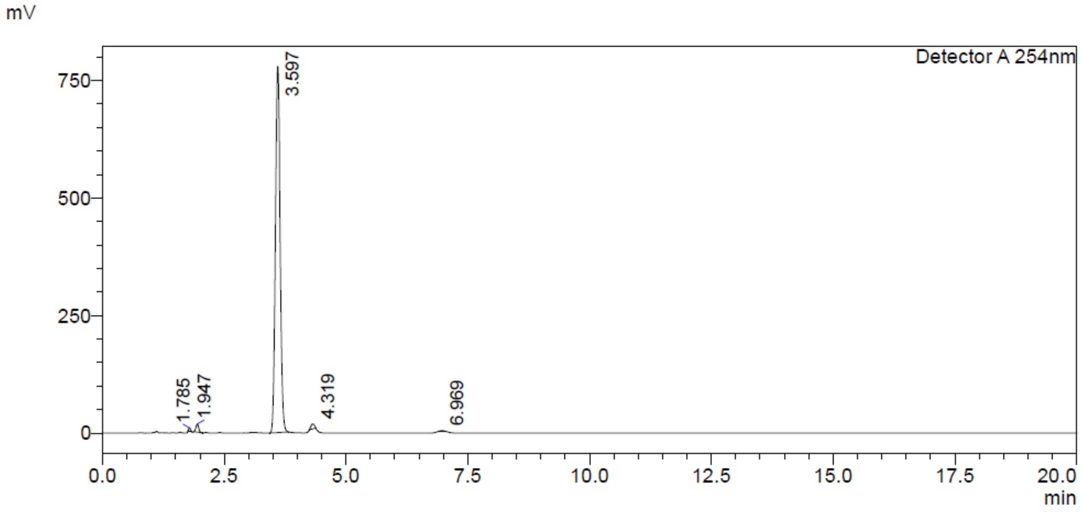

<Peak Table>

Detector A 254nm

Peak#	Ret. Time	Area	Height	Area%	Area/Height	Width at 5% Height
1	1.785	14974	6156	0.276	2.432	--
2	1.947	70976	17391	1.308	4.081	0.136
3	3.597	5281598	777539	97.342	6.793	0.223
4	4.319	52453	10665	0.967	4.918	--
5	6.969	5834	1195	0.108	4.880	--
Total		5425835	812946	100.000		

Figure **S8**. HPLC trace of **mCP-Br**.

mCP-Br$_2$

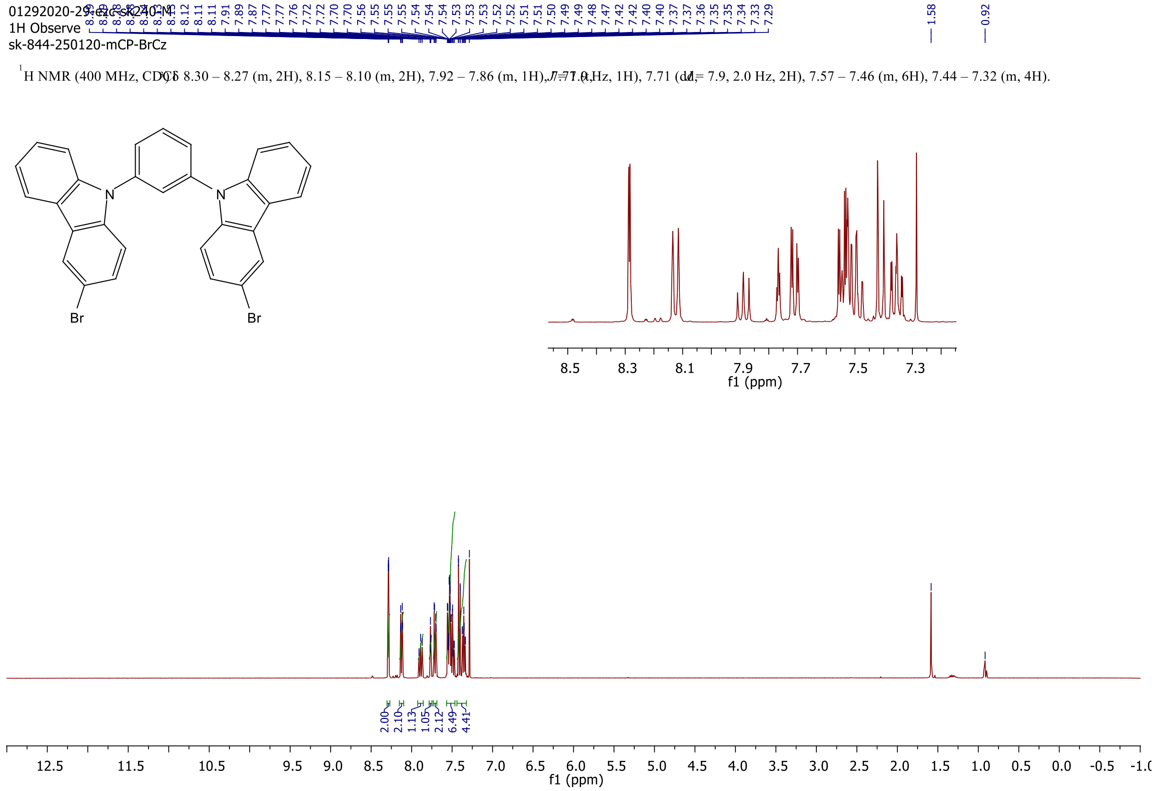

Figure **S9**. ^{1}H NMR of **mCP-Br$_2$** in $CDCl_3$.

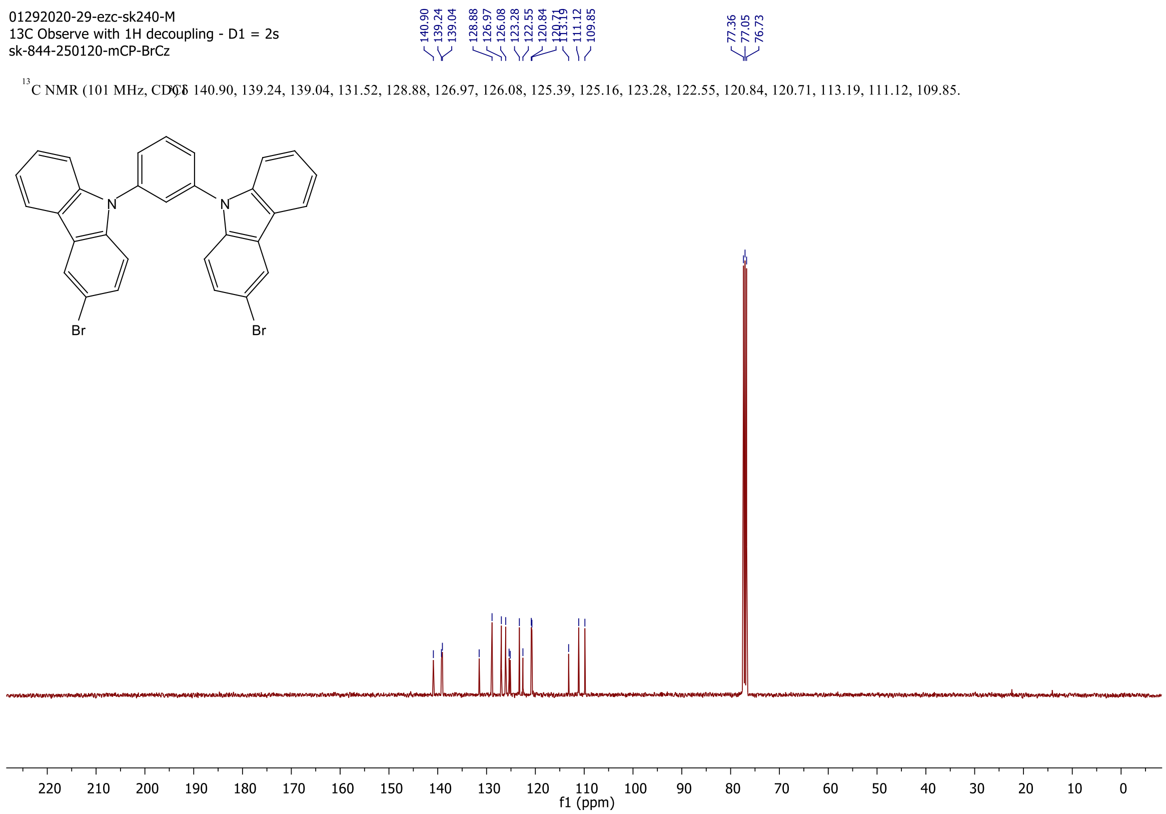

Figure **S10**. $^{13}C\{^{1}H\}$ NMR of **mCP-Br$_2$** in $CDCl_3$.

Display Report

Analysis Info

Analysis Name	D:\Data\Alans Data Aug 2023\STA_Choudhary 16003 (020823) DCMCPBR2.d	Acquisition Date	8/2/2023 2:32:00 PM
Method	AAAlansVGD2tune060723_low.m	Operator	demo
Sample Name	Choudhary 16003 (020823) DCMCPBR2	Instrument	micrOTOF 8213750.10408
Comment	Choudhary 16003 (020823) DCMCPBR2		

Acquisition Parameter

Source Type	ESI	Ion Polarity	Positive	Set Nebulizer	0.4 Bar
Focus	Not active			Set Dry Heater	200 °C
Scan Begin	50 m/z	Set Capillary	3500 V	Set Dry Gas	7.0 l/min
Scan End	3000 m/z	Set End Plate Offset	-500 V	Set Divert Valve	Waste

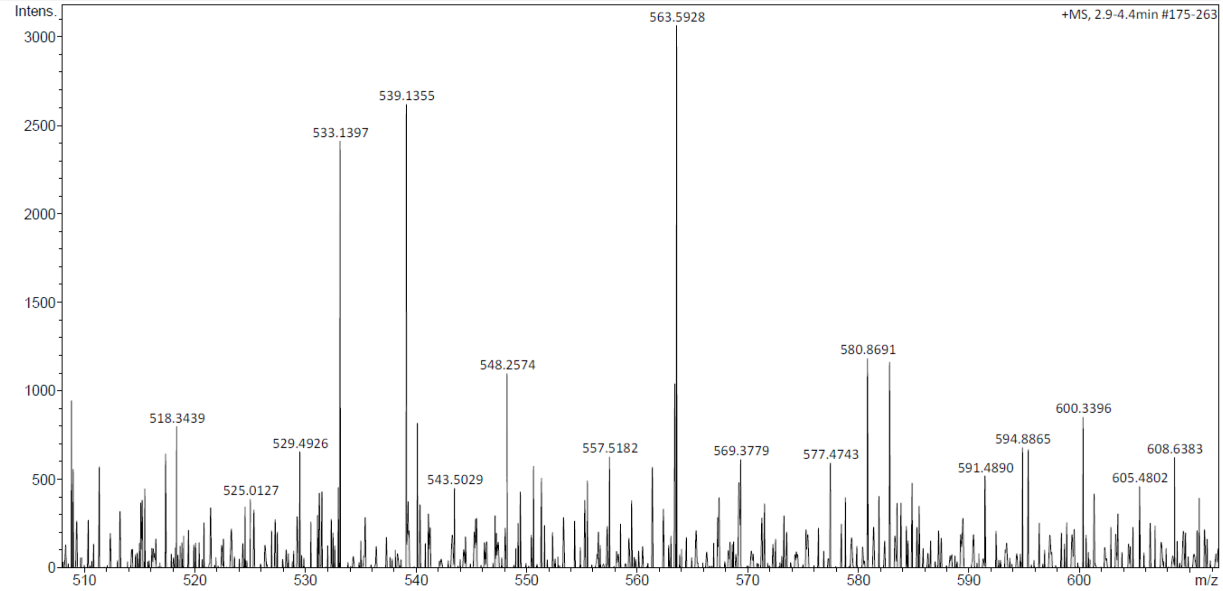

Bruker Compass DataAnalysis 4.1 printed: 8/3/2023 10:25:29 AM by: demo 1 of 1

Figure **S11**. HRMS of **mCP-Br$_2$**.

HPLC Trace Report19Jun2023

<Sample Information>

Sample Name	: dc-mCPBrCz		
Sample ID	:		
Method Filename	: 100% Methanol 20 mins.lcm		
Batch Filename	: mCP-BrCz		
Vial #	: 1-11	Sample Type	: Unknown
Injection Volume	: 5 uL		
Date Acquired	: 17/06/2023 15:48:28	Acquired by	: System Administrator
Date Processed	: 17/06/2023 17:03:31	Processed by	: System Administrator

<Chromatogram>

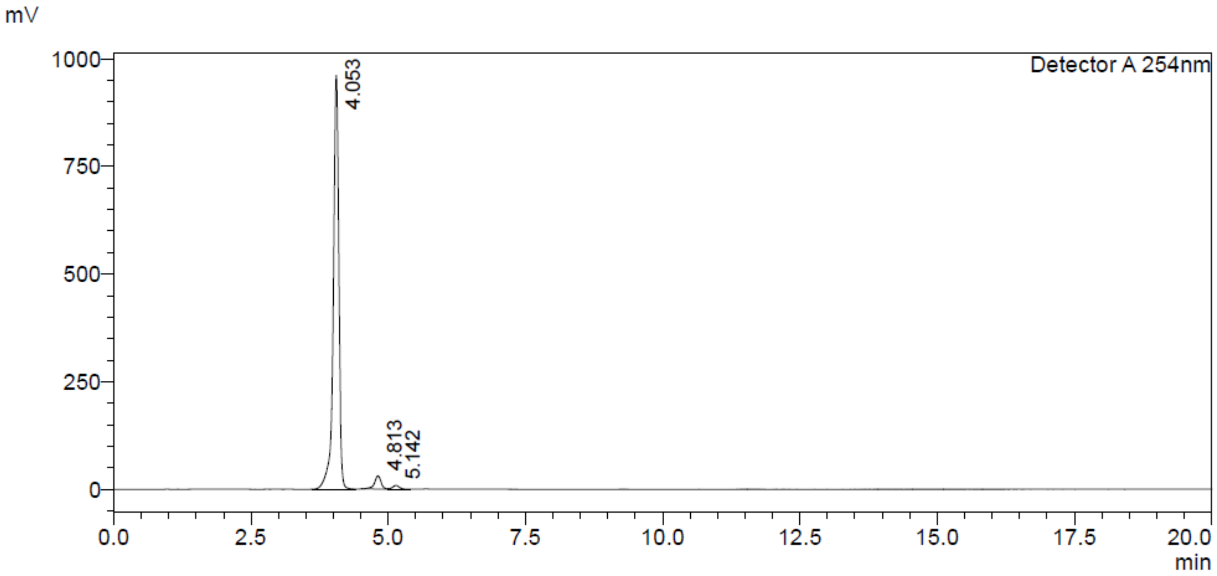

<Peak Table>

Detector A 254nm

Peak#	Ret. Time	Area	Height	Area%	Area/Height	Width at 5% Height
1	4.053	6691813	958328	95.409	6.983	0.273
2	4.813	253629	30496	3.616	8.317	0.332
3	5.142	68358	8377	0.975	8.160	--
Total		7013800	997201	100.000		

Figure **S12**. HPLC Trace of **mCP-Br_2**.

mCP-Br3

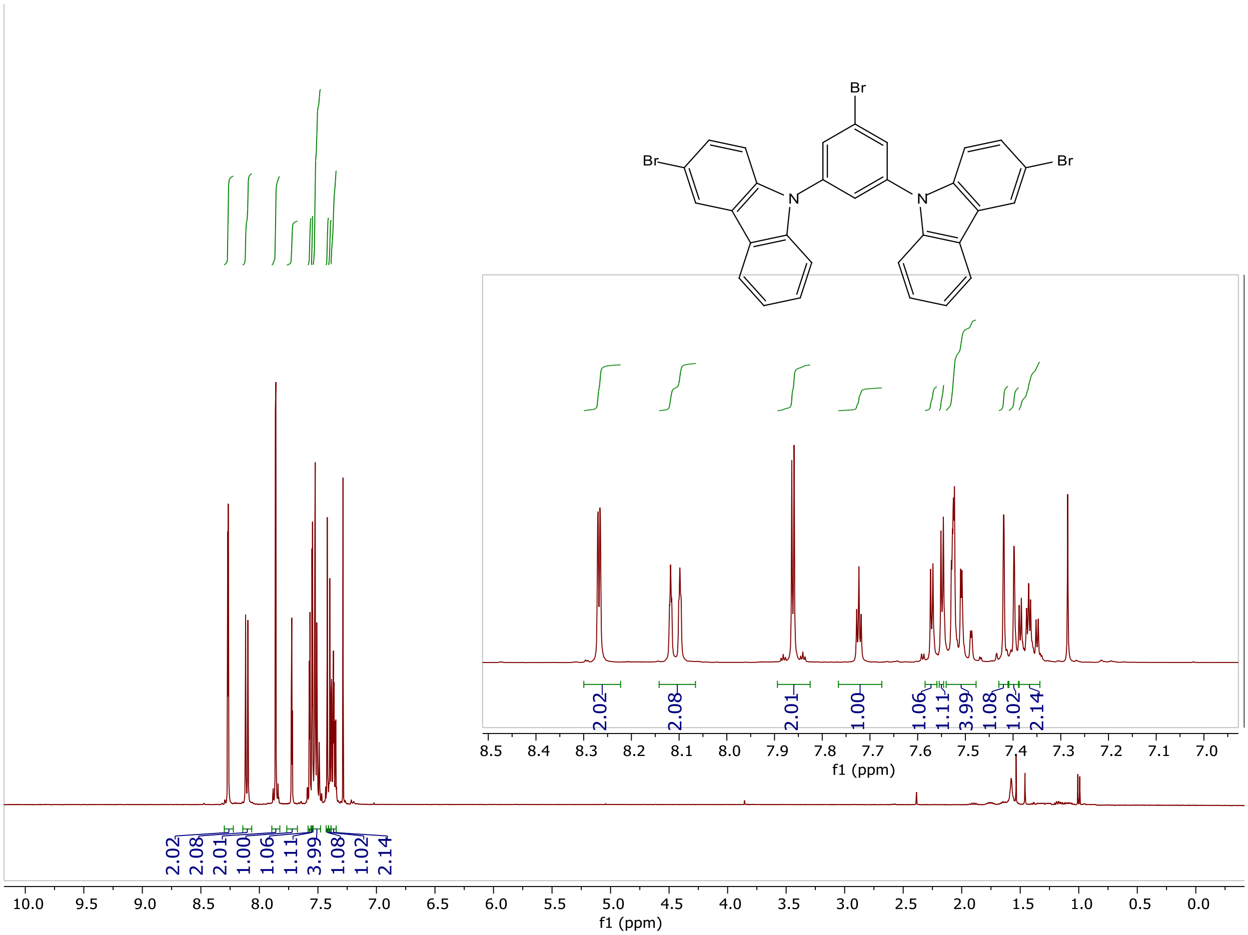

Figure **S13**. ^{1}H NMR of **mCP-Br$_3$** in $CDCl_3$.

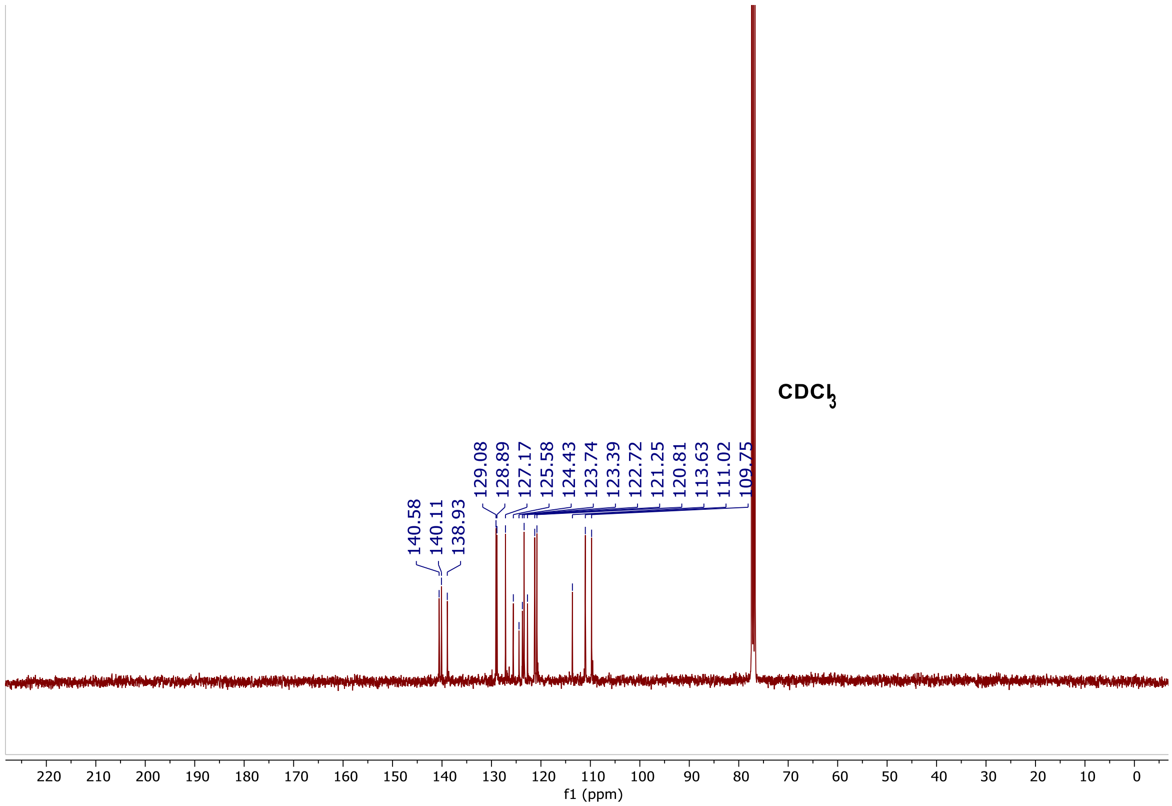

Figure **S14**. $^{13}C\{^{1}H\}$ NMR of **mCP-Br$_3$** in $CDCl_3$.

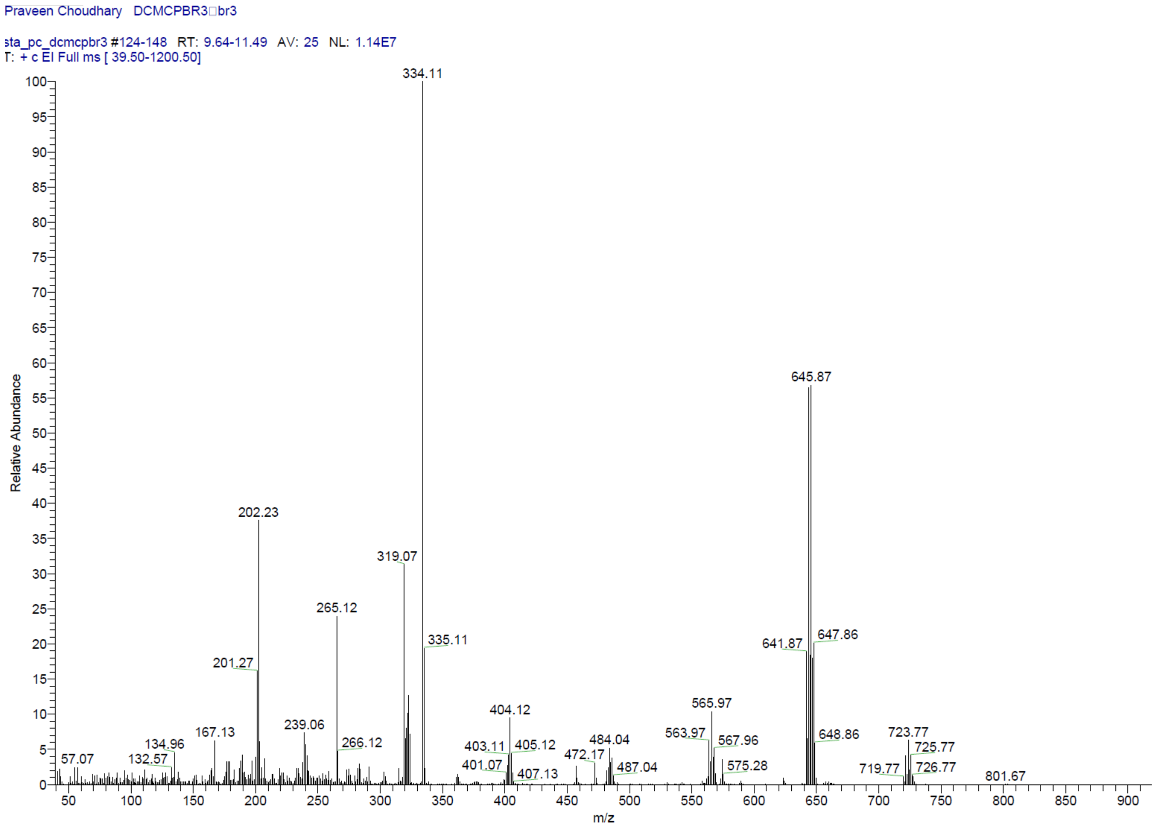

Figure **S15**. HRMS of **mCP-Br$_3$**.

HPLC Trace Report18Jun2023

<Sample Information>

Sample Name	: DC-BrmCPBrCz		
Sample ID	:		
Method Filename	:100% Acetonitrile 20 mins.lcm		
Batch Filename	: ALL.lcb		
Vial #	: 1-53	Sample Type	: Unknown
Injection Volume	: 10 uL		
Date Acquired	: 30/05/2023 15:33:31	Acquired by	: System Administrator
Date Processed	: 30/05/2023 15:53:33	Processed by	: System Administrator

<Chromatogram>

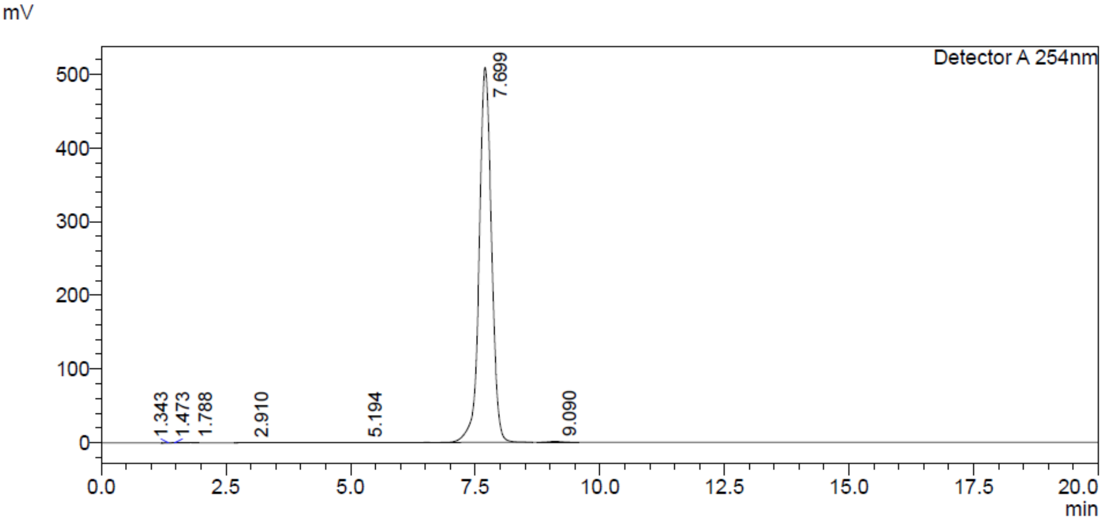

<Peak Table>

Detector A 254nm

Peak#	Ret. Time	Area	Height	Area%	Area/Height	Width at 5% Height
1	1.343	1925	257	0.022	7.481	--
2	1.473	1045	177	0.012	5.910	--
3	1.788	2643	156	0.030	16.982	--
4	2.910	3133	280	0.036	11.202	0.341
5	5.194	1495	126	0.017	11.836	0.349
6	7.699	8740085	508263	99.661	17.196	0.603
7	9.090	19469	1025	0.222	18.998	0.600
Total		8769795	510284	100.000		

Figure **S16**. HPLC trace of **mCP-Br$_3$**.

mCP-Br$_5$

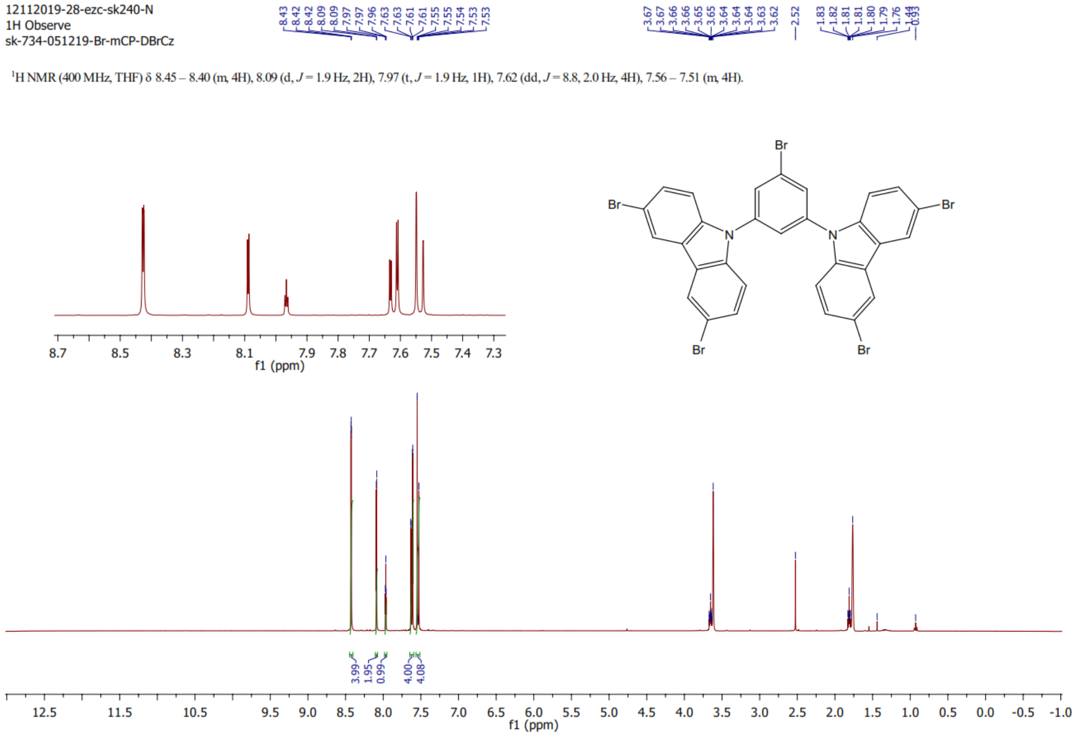

Figure **S17**. ^{1}H NMR of **mCP-Br$_5$** in THF-d_8.

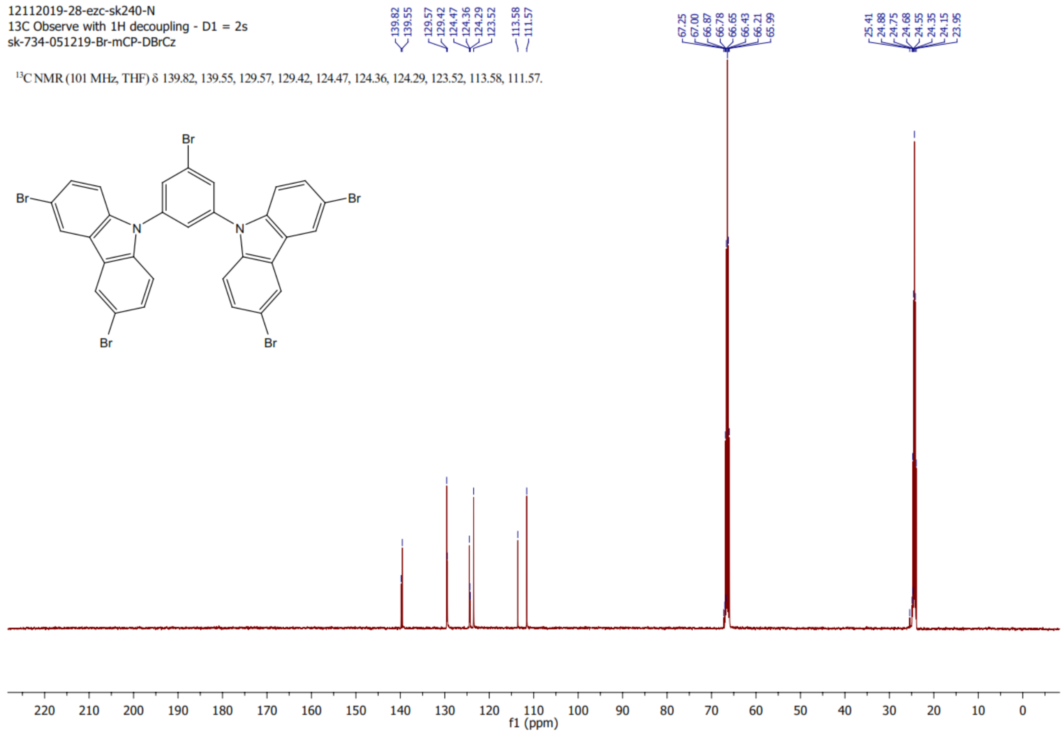

Figure **S18**. $^{13}C\{^{1}H\}$ NMR of **mCP-Br$_5$** in THF-d_8.

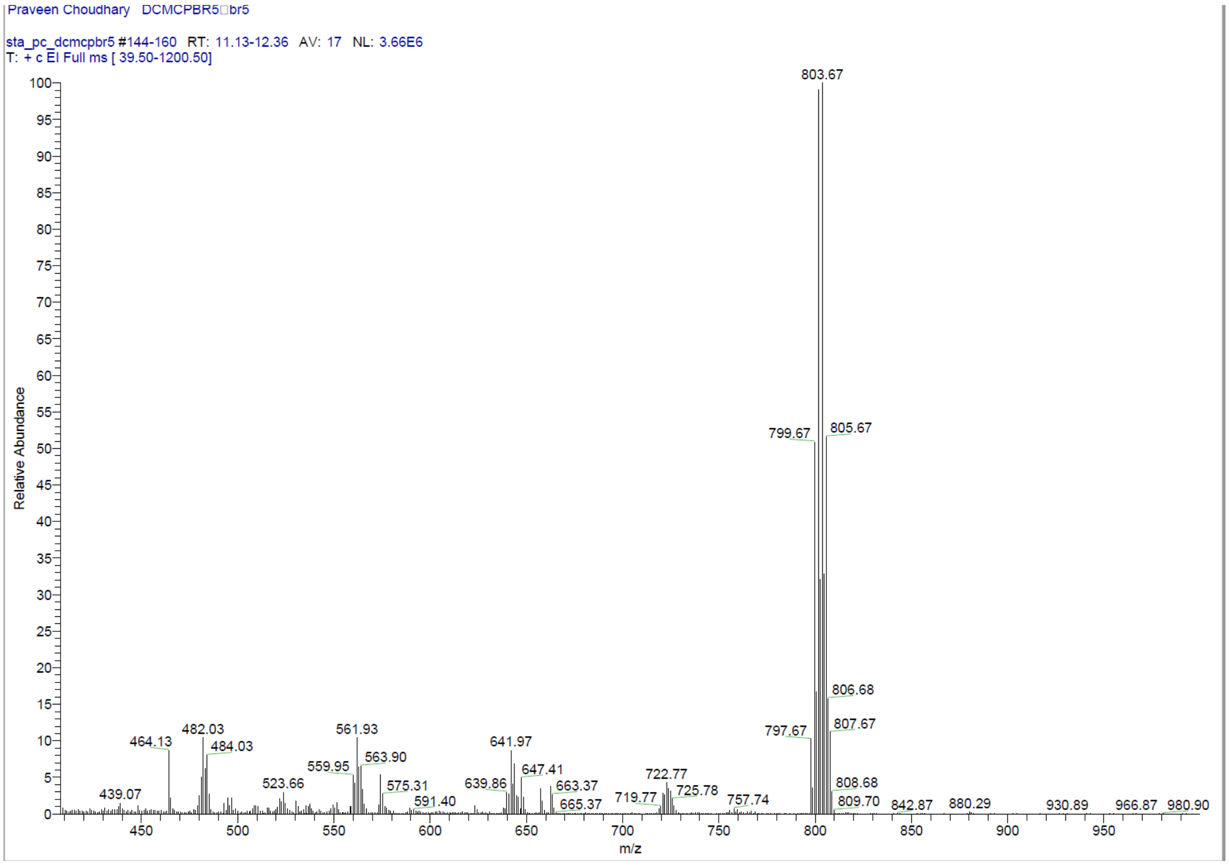

Figure **S19**. HRMS of **mCP-Br$_5$**.

HPLC Trace Report19Jun2023

<Sample Information>

Sample Name	: Br-mCP-DBrCz
Sample ID	: SK-734
Method Filename	: 100% Methanol B 10 mins.lcm
Batch Filename	: 05-03-2020.lcb
Vial #	: 1-6
Injection Volume	: 5 uL
Date Acquired	: 06/03/2020 17:46:15
Date Processed	: 19/06/2023 19:13:44

Sample Type	: Unknown
Acquired by	: System Administrator
Processed by	: System Administrator

<Chromatogram>

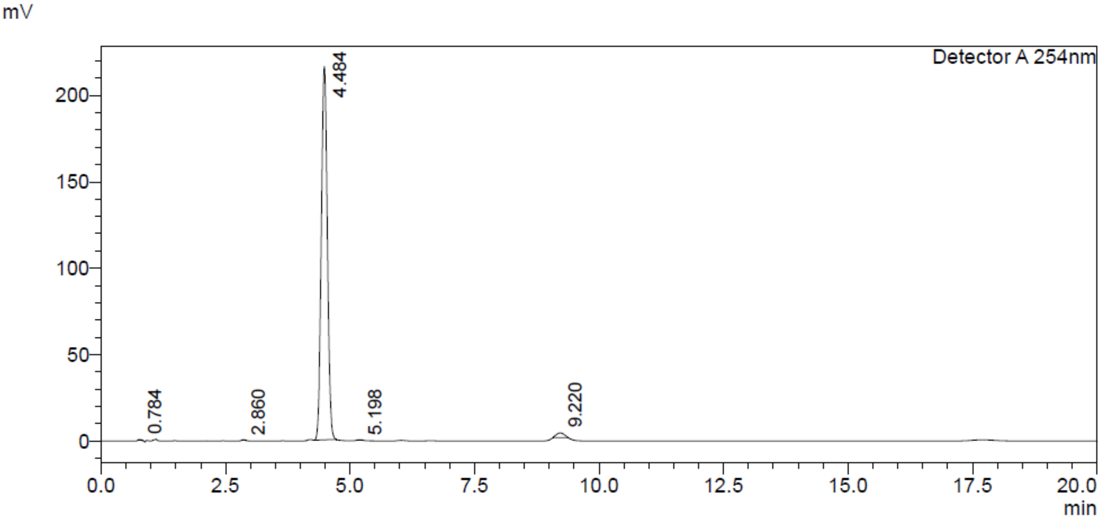

<Peak Table>

Detector A 254nm

Peak#	Ret. Time	Area	Height	Area%	Area/Height	Width at 5% Height
1	0.784	989	306	0.054	3.229	--
2	2.860	109	15	0.006	7.082	--
3	4.484	1800239	215104	98.324	8.369	0.276
4	5.198	797	190	0.044	4.193	--
5	9.220	28790	2641	1.572	10.902	0.288
Total		1830924	218256	100.000		

Figure **S20**. HPLC trace of **mCP-Br$_5$**.

Temperature-dependent PL

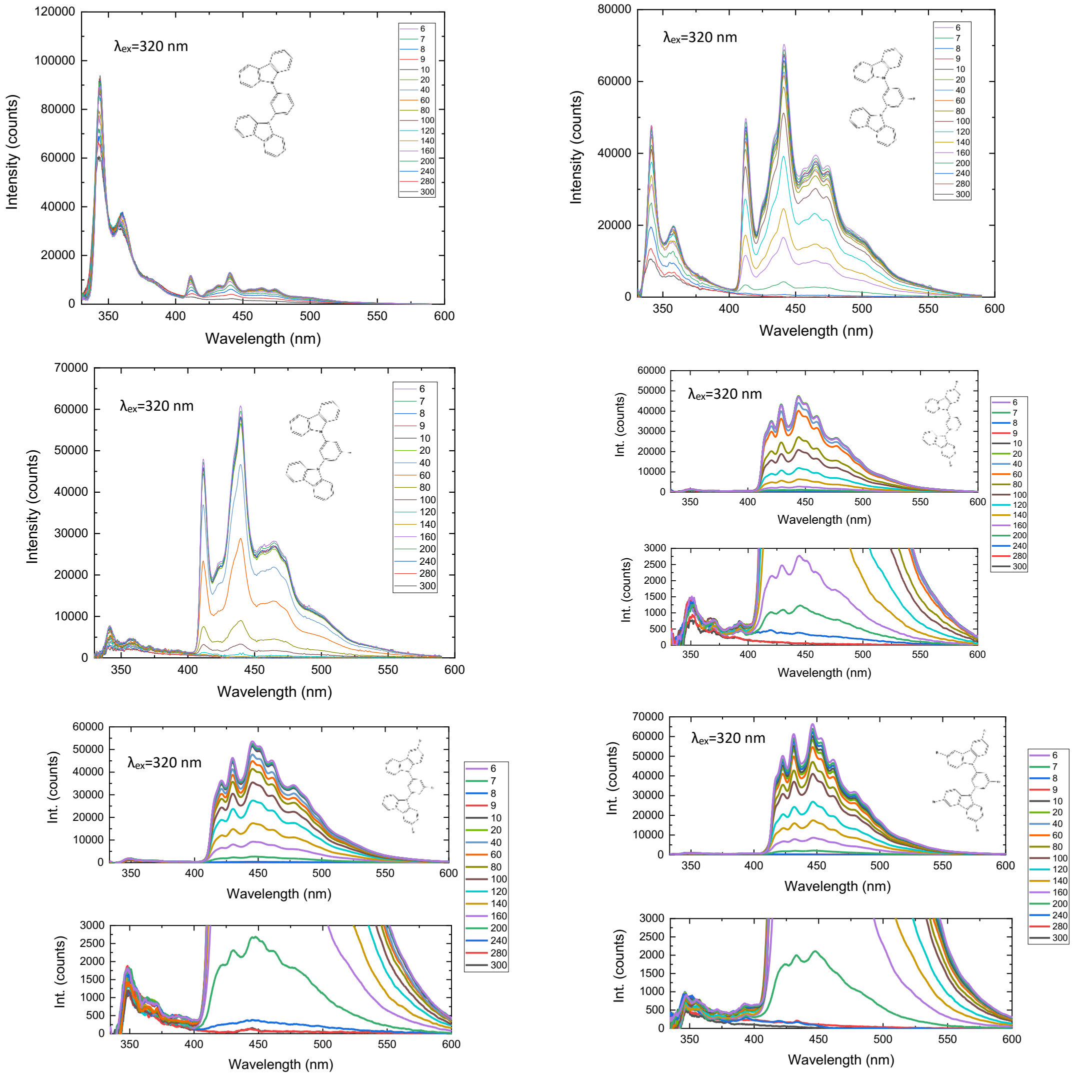

Figure **S21**. Temperature dependence of the photoluminescence of mCP and halogenated derivatives from 300 K to 6 K excited at 320 nm.

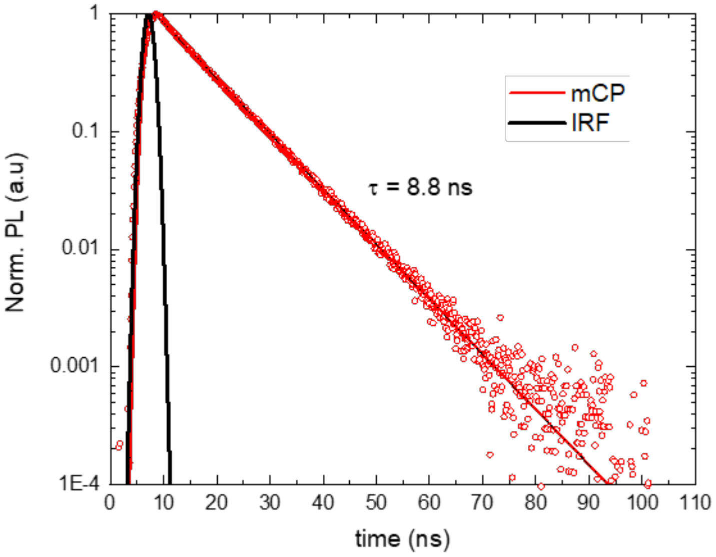

Figure **S22**. Transient fluorescence (measured at 360 nm) of a drop cast film of 0.1 wt.% mCP doped in PMMA at 6 K (λ_{exc} = 315 nm).

a)

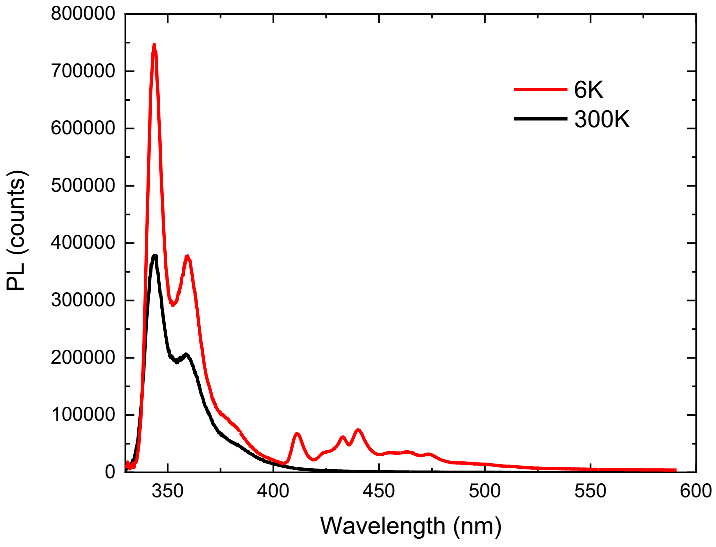

b)

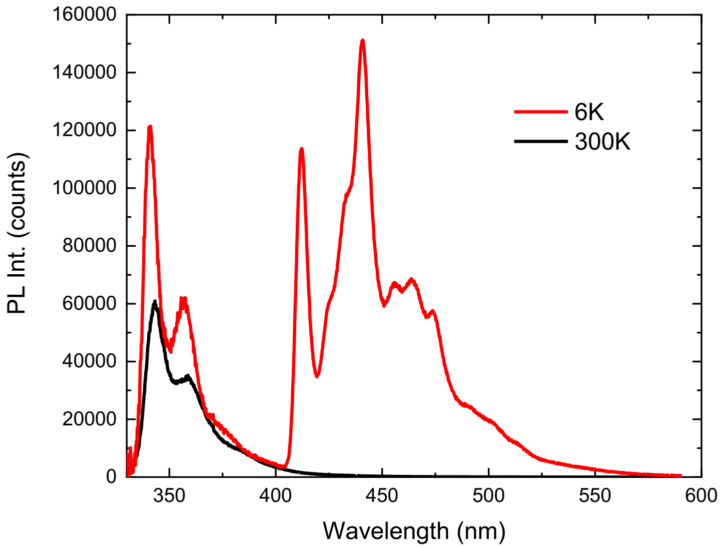

c)

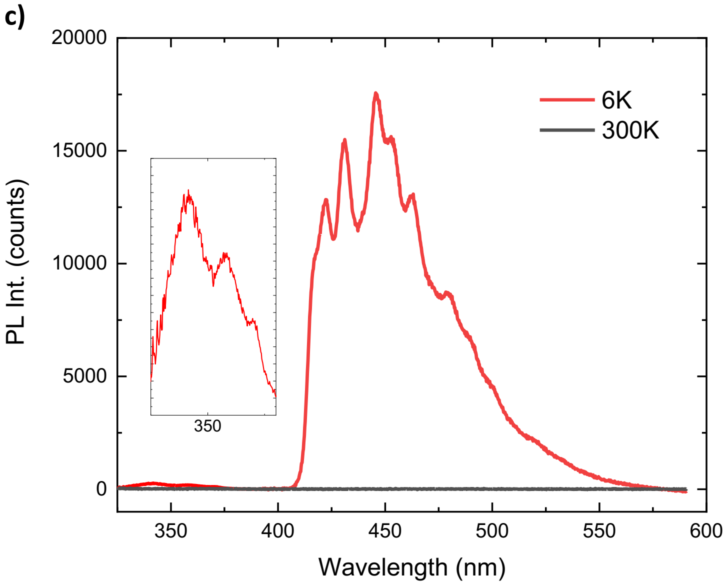

Figure **S23**. Photoluminescence at 6 K and 300 K, which shows fluorescence (330 - 400 nm) and phosphorescence (400 - 600 nm) emission of a drop cast film of 0.1 wt% a) mCP, b) mCP-Br and c) mCP-Br_5, doped in PMMA, excited at 315 nm. The observed trends and features are the same as for the 1 wt.% film.

Fit parameters

Table **S1**. Biexponential decay fit parameters of the transient PL decay of 1 wt.% mCP derivatives doped in PMMA thin films at 6 K with emission wavelength at 360 nm.

	$\mathbf{A_1\, e^{-\frac{t}{\tau_1}} + (1 - A_1)\, e^{-\frac{t}{\tau_2}}}$			$\tau_f = \frac{\sum A_i \tau_i^2}{\sum A_i \tau_i}$
	A_1	τ_1 (ns)	τ_2(ns)	τ_f (ns)
**mCP**	0.52 ± 0.03	2.2 ± 0.1	6.9 ± 0.1	5.7 ± 0.1
**mCP-Br**	0.890 ± 0.006	1.06 ± 0.02	3.76 ± 0.08	1.88 ± 0.05
**mCP-I**	0.973 ± 0.003	0.34 ± 0.02	3.5 ± 0.1	1.04 ± 0.07
**mCP-Br$_2$**	0.68 ± 0.02	0.047 ± 0.004	0.284 ± 0.006	0.222 ± 0.07
**mCP-Br$_3$**	0.81 ± 0.04	0.072 ± 0.006	0.21 ± 0.02	0.128 ± 0.01
**mCP-Br$_5$**	0.979 ± 0.002	0.0152 ± 0.0006	0.229 ± 0.009	0.067 ± 0.005

Table **S2**. Biexponential decay fit parameters of the transient PL decay of 10 wt.% TPA-DCPP thin films doped in mCP and mCP derivatives with emission wavelength at 630 nm.

	$\mathbf{A_1\, e^{-\frac{t}{\tau_1}} + (1 - A_1)\, e^{-\frac{t}{\tau_2}}}$			$\tau_d = \frac{\sum A_i \tau_i^2}{\sum A_i \tau_i}$
	A_1	τ_1 (μs)	τ_2(μs)	τ_d (μs)
**mCP**	0.97 ± 0.01	31 ± 2	118 ± 4	40 ± 3
**mCP-Br**	0.970 ± 0.003	31 ± 1	123 ± 1	41 ± 1
**mCP-I**	0.983 ± 0.001	29.7 ± 0.2	101.7 ± 0.8	33.7 ± 0.3
**mCP-Br$_2$**	0.973 ± 0.002	29.2 ± 0.3	91.8 ± 0.6	34.2 ± 0.4
**mCP-Br$_3$**	0.976 ± 0.002	27.9 ± 0.3	79.6 ± 0.6	31.3 ± 0.04
**mCP-Br$_5$**	0.976 ± 0.003	27.3 ± 0.4	79.6 ± 0.9	30.8 ± 0.5

Ground state geometry optimization

Table **S3.** Summary of bond lengths and dihedral angles at the S_0 optimized geometry of the halogenated mCP molecules obtained at CAM-B3LYP/6-31+g(d,p) level.

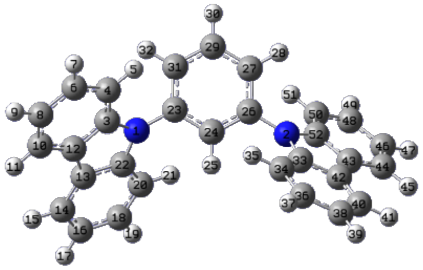

	d_{29-30} (Å)	d_{8-9} (Å)	d_{16-17} (Å)	$\Theta_{31-23-1-22}$ (°)	$\Theta_{27-26-2-33}$ (°)
**mCP**	1.09	1.09	1.09	120.0	120.0
**mCP-Br**	1.94	1.09	1.09	122.9	122.9
**mCP-I**	2.12	1.09	1.09	122.2	122.2
**mCP-Br_2**	1.09	1.09	1.95	119.9	119.9
**mCP-Br_3**	1.94	1.95	1.95	121.7	121.7
**mCP-Br_5**	1.94	1.95	1.95	122.0	122.0

TD-DFT calculation

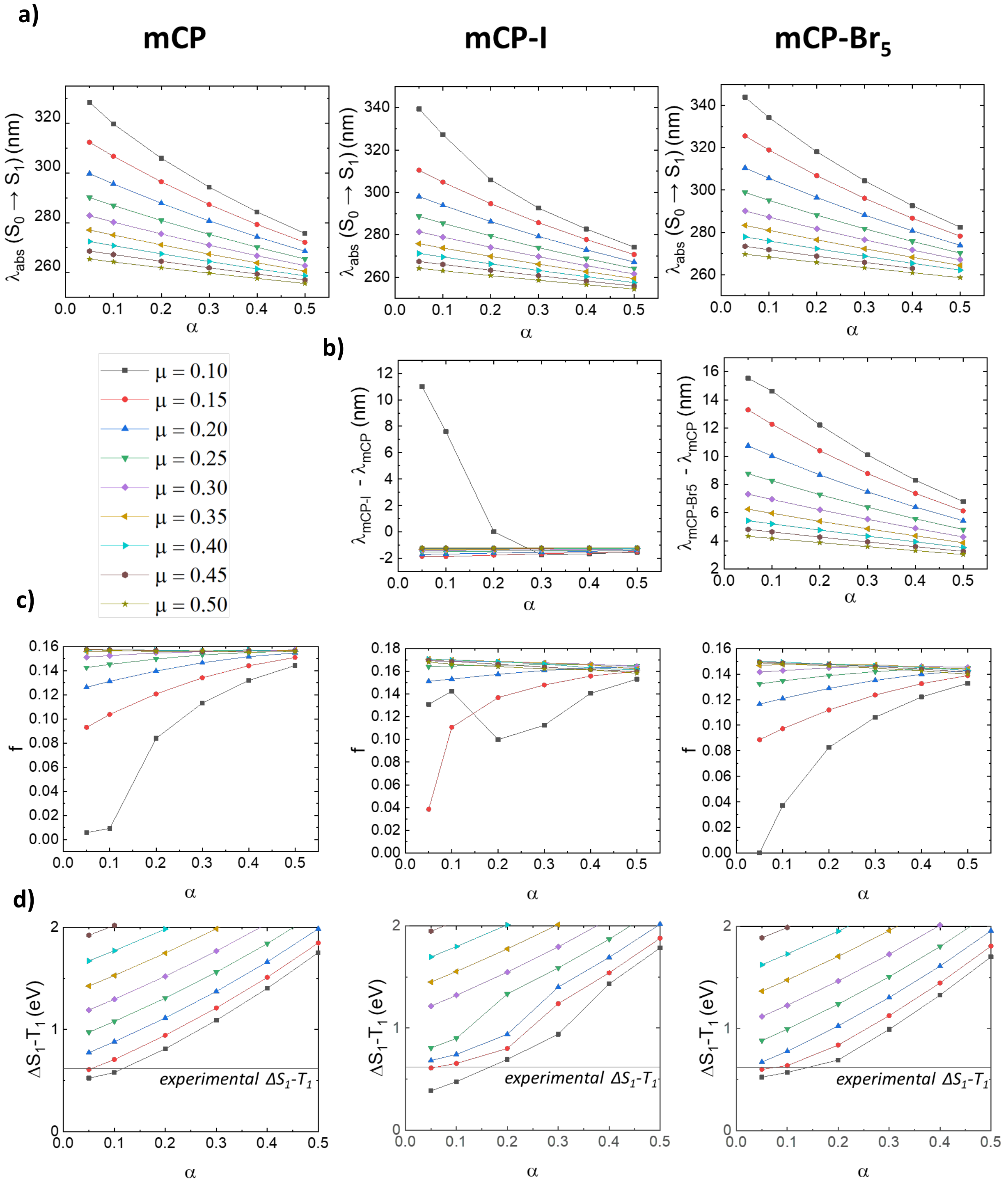

Figure **S24**. TD-DFT calculation of a) absorption wavelength of $S_0 \rightarrow S_1$, b) spectral shift of the absorption wavelength induced by halogenation, c) oscillator strength and d) energy difference between S_1 and T_1 for (from left to right) mCP, mCP-I and mCP-Br_5 using CAM-B3LYP/6-31+g** with different tuning parameters $(\mu, \alpha, \beta = 1 - \alpha)$.

Absorption and PL spectra of TPA-DCPP in mCP derivatives

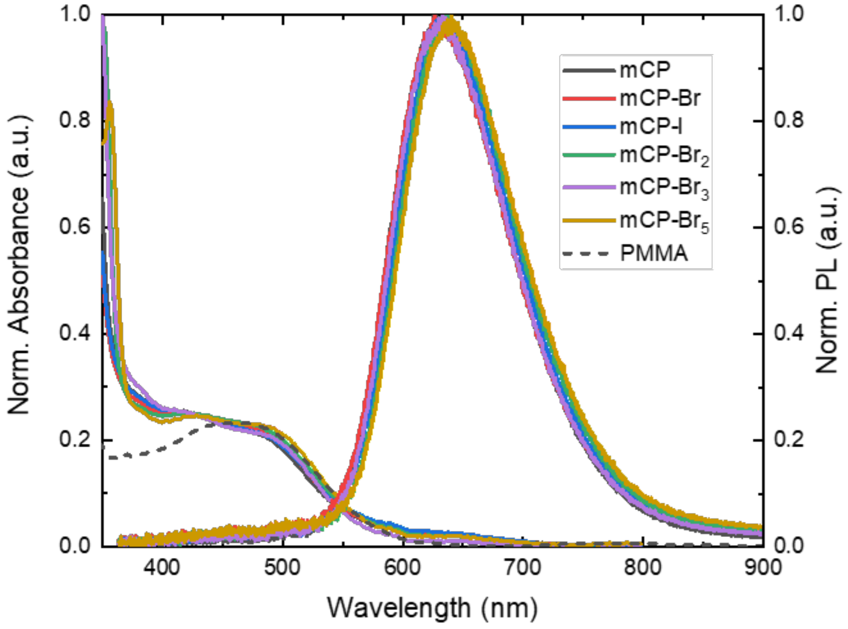

Figure **S25.** Normalized absorption (left) and emission (right) spectra of 10 wt.% TPA-DCPP in mCP derivatives. The dashed line shows the absorption of 10 wt.% TPA-DCPP in PMMA to distinguish it from that of the mCP hosts.

RISC rate of 4CzIPN doped films

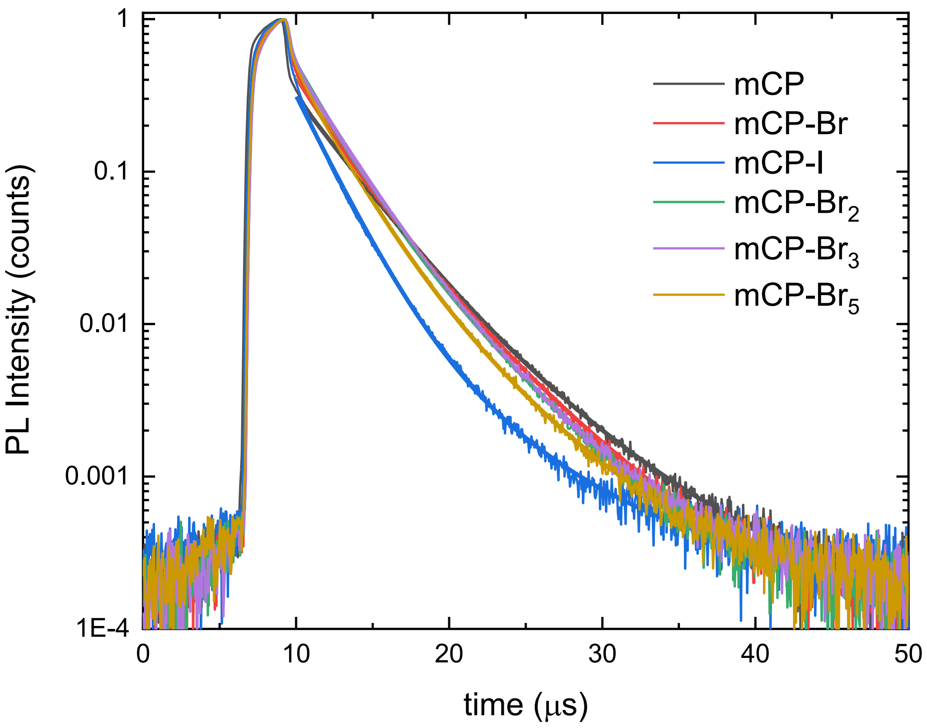

Figure **S26**. Transient PL of doped thin films of 10 wt.% 4CzIPN in different halogen mCP hosts excited at 400 nm (emission at 550 nm). Fits corresponds to delayed fluorescence part of the emission.

Table **S4**. Biexponential decay fit parameters of the transient PL decay of 10 wt.% 4CzIPN thin films doped in mCP and mCP derivatives with emission wavelength at 550 nm.

	$\mathbf{A_1\, e^{-\frac{t}{\tau_1}} + (1 - A_1)\, e^{-\frac{t}{\tau_2}}}$			$\boldsymbol{\tau_d = \frac{\sum A_i \tau_i^2}{\sum A_i \tau_i}}$
	A_1	τ_1 (μs)	τ_2(μs)	τ_d (μs)
**mCP**	0.974 ± 0.008	3.1 ± 0.1	6.2 ± 0.3	3.3 ± 0.1
**mCP-Br**	0.967 ± 0.007	2.5 ± 0.1	4.9 ± 0.1	2.65 ± 0.1
**mCP-I**	0.997 ± 0.001	2.08 ± 0.06	5.7 ± 0.3	2.11 ± 0.06
**mCP-Br$_2$**	0.973 ± 0.007	2.3 ± 0.1	4.5 ± 0.1	2.41 ± 0.09
**mCP-Br$_3$**	0.98 ± 0.01	2.5 ± 0.1	5.1 ± 0.2	2.6 ± 0.1
**mCP-Br$_5$**	0.987 ± 0.009	2.30 ± 0.09	5.0 ± 0.2	2.4 ± 0.1

Table **S5**. Summary of photophysical properties and calculated RISC rate of doped thin films of 10 wt.% of 4CzIPN in different mCP analogues.

Host	τ_d (μs)	Φ (%)	Φ_p (%)	Φ_d (%)	k_{RISC} (10^6 s^{-1})
mCP	3.3 ± 0.1	64 ± 3	14.7 ± 0.4	49 ± 3	1.18 ± 0.09
mCP-Br	2.7 ± 0.1	61 ± 3	7.3 ± 0.2	54 ± 3	3.0 ± 0.2
mCP-I	2.11 ± 0.06	58 ± 3	7.5 ± 0.2	50 ± 3	3.4 ± 0.2
mCP-Br$_2$	2.41 ± 0.09	67 ± 3	6.0 ± 0.2	61 ± 3	4.5 ± 0.3
mCP-Br$_3$	2.6 ± 0.1	66 ± 3	5.9 ± 0.2	60 ± 3	4.2 ± 0.3
mCP-Br$_5$	2.4 ± 0.1	61 ± 3	6.1 ± 0.2	55 ± 3	4.0 ± 0.3

(1) Wong, M. Y.; Hedley, G. J.; Xie, G.; Kölln, L. S.; Samuel, I. D. W.; Pertegás, A.; Bolink, H. J.; Zysman-Colman, E. Light-Emitting Electrochemical Cells and Solution-Processed Organic Light-Emitting Diodes Using Small Molecule Organic Thermally Activated Delayed Fluorescence Emitters. *Chem. Mater.* **2015**, *27* (19), 6535–6542. https://doi.org/10.1021/acs.chemmater.5b03245.

(2) O'Driscoll, L. J.; Wang, X.; Jay, M.; Batsanov, A. S.; Sadeghi, H.; Lambert, C. J.; Robinson, B. J.; Bryce, M. R. Carbazole-Based Tetrapodal Anchor Groups for Gold Surfaces: Synthesis and Conductance Properties. *Angew. Chemie Int. Ed.* **2020**, *59* (2), 882–889. https://doi.org/10.1002/ANIE.201911652.

(3) Park, S. R.; Kim, S. M.; Kang, J. H.; Lee, J. H.; Suh, M. C. Bipolar Host Materials with Carbazole and Dipyridylamine Groups Showing High Triplet Energy for Blue Phosphorescent Organic Light Emitting Diodes. *Dye. Pigment.* **2017**, *141*, 217–224. https://doi.org/10.1016/J.DYEPIG.2017.02.014.

(4) Chen, F.; Liu, N.; Ji, E.; Dai, B. Copper/β-Diketone-Catalysed N-Arylation of Carbazoles. *RSC Adv.* **2015**, *5* (64), 51512–51523. https://doi.org/10.1039/C5RA07690K.
